# Mapping Habitat Suitability of the Asiatic Black Bear (*Ursus thibetanus*) in the Kalam Valley, Pakistan: An Ensemble Modelling Approach

**DOI:** 10.3390/ani16193080

**Published:** 2026-09-30

**Authors:** Arshad Ali, Abdur Rahim, Muhammad Farooq, Luciano Bosso

**Affiliations:** 1 Department of Zoology, University of Malakand, Chakdara 18000, Pakistan; drrahimkhan@uom.edu.pk; 2 Department of Zoology, Wildlife and Fisheries, PMAS-Arid Agriculture University, Rawalpindi 46300, Pakistan; farooq378@gmail.com; 3 Institute for Research on Innovation, International Law and Sustainability (IRIDIS), National Research Council of Italy, Via Cardinale Guglielmo Sanfelice 8, 80134 Napoli, Italy

**Keywords:** Asiatic black bear, ensemble modelling, habitat suitability, Kalam Valley, Pakistan, *Ursus thibetanus*

## Abstract

The Asiatic black bear (*Ursus thibetanus*), a large and vulnerable mammal in northern Pakistan, faces increasing threats from habitat loss, tourism, human disturbance, and human–bear interactions. However, spatially explicit information on its potential habitat remains limited. In this study, 126 new Asiatic black bear presence records, collected through bear sign surveys and camera trapping in the Kalam Valley, were combined with an ensemble modelling approach incorporating regression-based, machine-learning, and maximum entropy models to map habitat suitability and identify key areas for conservation planning in northern Pakistan. The resulting ensemble habitat suitability map indicated relatively higher suitability in the northern and north-central portions of the study area, whereas southern areas consistently exhibited lower relative suitability. The binary suitability map classified 1061.22 km^2^ as suitable habitat, mainly located between 400 and 3500 m above sea level. Variable importance analysis identified the presence of rivers as the most important predictor of habitat suitability, followed by distance to settlements and the vector ruggedness measure. All models showed high values of the AUC, TSS, and Boyce index. Our results provide a spatially explicit and practical basis for habitat protection, field validation, connectivity assessment, and conflict-sensitive conservation planning for the Asiatic black bear in northern Pakistan.

## Simple Summary

The Asiatic black bear (*Ursus thibetanus*) is a vulnerable species in northern Pakistan, threatened by habitat loss and fragmentation, tourism, and conflicts with local communities. However, information on where suitable habitats remain is still limited. About 126 bear records, obtained from field surveys and camera traps, were used to produce habitat suitability models to identify areas that could support the species. A suitable habitat area of 1061 km^2^ was identified in the Kalam Valley. Rivers were the most important factor influencing bear presence, followed by distance to settlements, landscape ruggedness, rainfall, and land cover. These findings can help guide habitat protection, connectivity conservation, and conflict reduction.

## 1. Introduction

Large carnivores depend on extensive home ranges and interconnected habitats to maintain viable populations [1,2,3]. However, the rapid expansion of human activities continues to drive the loss, degradation, and fragmentation of natural environments, posing a serious threat to the conservation of these species [4,5]. As a result, many carnivore populations have declined sharply and now face an increasing risk of global extinction [6,7]. Effective conservation strategies must therefore prioritise the identification and protection of suitable habitats, the preservation of ecosystem functionality, and the systematic evaluation of habitat quality to support evidence-based wildlife management [8].

The Asiatic black bear (*Ursus thibetanus*) is classified as “vulnerable” on the International Union for Conservation of Nature (IUCN) Red List [9] and is included in Appendix I of the “Convention on International Trade in Endangered Species of Wild Fauna and Flora” [10]. The species is distributed across Pakistan, Afghanistan, Iran, India, Japan, Indochina, Korea, Laos, Vietnam, and Taiwan [9,11,12]. It is a key large carnivore in Asia and is capable of long-distance dispersal [13]. It has a patchy distribution, extending from southeastern Iran to Japan, Russia, Korea, the Indo-Chinese Himalayan region, and parts of Southeast Asia [9,14,15,16]. Despite its broad range, the species persists across the landscape, where topography, forest structure, climate, and human disturbances interact to shape habitat availability and connectivity [17].

In Pakistan, the Asiatic black bear is distributed primarily in the forested landscape of Khyber Pakhtunkhwa (Swat, Dir, Siran Valleys, and Battagram), Azad Jammu and Kashmir (Neelum and Kaghan), and lower-elevation areas of Gilgit Baltistan, where it prefers coniferous, mixed coniferous, and deciduous forests abundant in oak, shrub, and grasses [9,17,18,19,20,21,22,23]. In northern Pakistan, the Asiatic black bear’s habitats and elevational ranges vary seasonally due to its dynamic, opportunistic nature, prompting it to move among habitats in search of food [24,25]. The Asiatic black bear population is threatened by habitat loss and fragmentation, illegal hunting, commercial poaching, food depletion, and the drastic shrinking of its distribution range [17,26]. At the same time, human–bear interactions remain an important practical concern, as habitat loss and human expansion increase spatial overlap and the likelihood of conflict in agro-forested valleys [27,28]. Several studies indicated that suitable habitats for Asiatic black bears were located throughout mountainous landscapes with dense understory vegetation, abundant food sources, rugged terrain, and sufficient denning sites [9,17]. Bears usually prefer steep southern slopes near rivers and trails [29] and tend to avoid rocky, unvegetated, and human-dominated areas where intense anthropogenic pressures, such as tourism growth, infrastructure development, forest resource extraction, and land-use changes, occur. This situation highlights the urgency of identifying bears’ critical habitat suitability and potential connectivity [30,31]. Despite growing research on Asiatic black bear ecology and modelling in northern Pakistan, field-based valley-scale mapping of critical habitat remains limited, even though the region’s complex forest-pasture mosaics can strongly influence habitat suitability predictions [21,31,32].

Effective conservation and management of the distribution and habitat of threatened large carnivores, especially with limited data, are essential for prioritizing conservation in the mountainous, human-dominated landscape [32,33,34,35,36]. Therefore, proper knowledge of the Asiatic black bear requires accurate identification of suitable habitat at meaningful ecological scales [31]. In conservation biology, the use of advanced statistical techniques, combined with Geographic Information Systems, Remote Sensing, Global Positioning System (GPS), and spatially explicit Habitat Suitability Modelling (HSM), is growing rapidly, helping to assess habitat suitability across species ranges [8]. To assess suitable habitat distribution, multiple models, such as mechanistic models [37], regression models [38], HSMs, and ecological niche models, have been used [39,40,41]. However, most HSM analyses have relied on individual models, whose predictive performance varies widely across methods and species [42]. The combined use of multiple models based on an ensemble modelling approach provides more robust estimates of potential species distributions and reduces uncertainty associated with reliance on a single algorithm [41,43,44]. Additionally, ensemble mapping emphasizes areas with consistent suitability, making them more reliable for conservation [42,43,44].

In this study, a combined approach of field surveys and HSM was used to identify the habitat suitability of the Asiatic black bear in the Kalam Valley and map a key core area for improving conservation strategies. It was hypothesised that habitat suitability would be highest in forested, productive areas with rugged terrain and would decline with increasing human disturbance and accessibility across the Kalam Valley [17]. These results, within a global conservation context, help prioritise habitats based on evidence, inform connectivity and land-use decisions in rapidly changing mountain valleys, and provide a clear spatial basis for aligning protected-area management and threat mitigation with the species’ ecological needs [17].

## 2. Materials and Methods

### 2.1. Study Area

**Figure 1:**
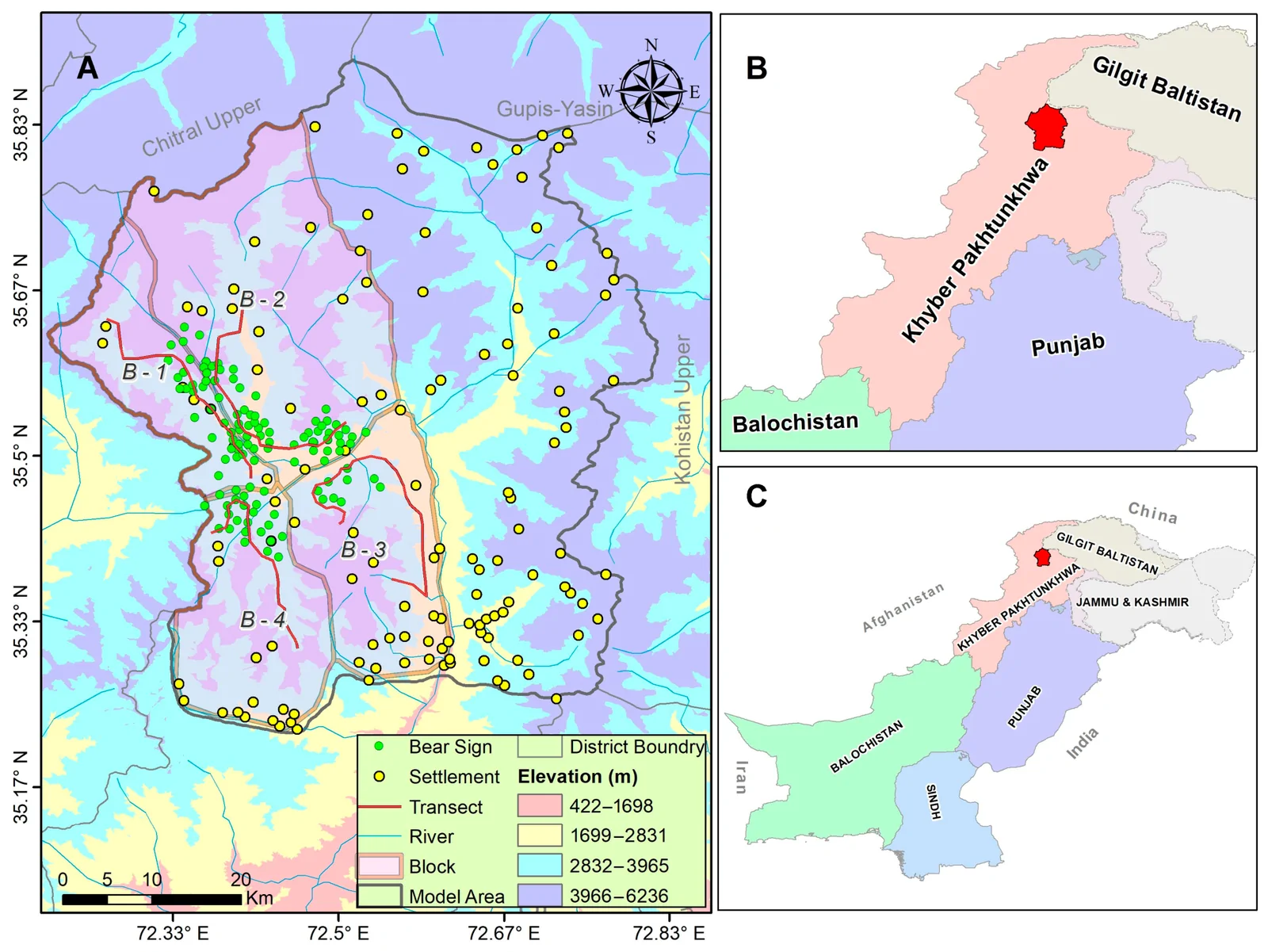
Field survey, modelled area, and elevation of the Kalam Valley study area (**A**), its location within the Khyber Pakhtunkhwa province (**B**), and within Pakistan (**C**). The study area is shown in red in both (**B**,**C**). B-1, B-2, B-3, and B-4 = Asiatic Black Bear sampling blocks.

This study focused on the Kalam Valley (Northern Pakistan), one of the important forested mountain systems within the Asiatic black bear range in Khyber Pakhtunkhwa province. The study area lies approximately between 35.17–35.83° N latitude and 72.33–72.83° E longitude (Figure 1). The Kalam Valley is located in the upper Swat District, approximately 99 km from the city of Mingora, and is widely known for its lakes, waterfalls, forested slopes, alpine pastures, and high scenic value.

The Kalam Valley elevation ranges from the low valley bottom at 422 m to high mountain terrain at 6236 m above sea level (a.s.l.). This pronounced topographic variation produces strong climatic, vegetation, and habitat gradients relevant to Asiatic black bear distribution [17]. The climate is harsh during winter, with frequent snowfall from November to February, whereas June to August is comparatively mild and favourable for tourism, grazing, and field access. Rainfall is more frequent in March and April, while summer and autumn are relatively drier [45]. These seasonal conditions are important because Asiatic black bears may shift their habitat use in response to food availability, snow cover, human activity, and access to forested habitats. The Kalam Valley is predominantly characterised by moist temperate coniferous forests, including silver fir (*Abies pindrow*), spruce (*Picea smithiana*), deodar (*Cedrus deodara*), and kail or blue pine (*Pinus wallichiana*). The rugged mountainous terrain provides habitat for populations of mountain ungulates, including the Kashmir markhor (*Capra falconeri cashmiriensis*) and the Himalayan ibex (*Capra ibex sibirica*) [31]. Furthermore, the valley supports a diverse array of other wildlife, including mammals such as musk deer *(Moschus chrysogaster*), markhor, and the rhesus monkey (*Macaca mulatta*); pheasants such as the monal pheasant (*Lophophorus impejanus*), Koklass pheasant (*Pucrasia macrolopha*), snow cock (*Tetraogallus himalayensis*), chukor partridge (*Alectoris chukar*), and see-see partridge (*Ammoperdix griseogularis*); and other wildlife [46]. The same forested and rugged landscape also provides shelter, feeding opportunities, denning sites, and movement corridors for the Asiatic black bear.

### 2.2. Presence Records

The study was conducted between May 2024 and September 2025 in the upper reaches of Kalam Valley, northern Pakistan (Figure 1). Presence records of the Asiatic black bear were collected by using bear sign surveys and camera trapping. Initially, a reconnaissance visit was conducted in May 2024 to obtain general information about the study area, identify potential bear habitats, and understand accessibility conditions for subsequent field surveys. Following this preliminary assessment, field surveys for Asiatic black bear presence were conducted during July–August 2024 and June–July 2025. For systematic sampling, the study area was divided into four sampling blocks (by plotting a 10 km-long transect in each block) based on the geographical extent, accessibility, elevation gradients, and major habitat variation of the valley. These blocks represented spatial units used to organize field surveys and ensure coverage of different landscape conditions within the study area. A total of four transects (one transect per block) were established. These were not straight-line transects; rather, they followed a loop-like design where possible to maximize coverage of available habitat types and elevation ranges within each block. The first two transects were surveyed during July–August 2024, while the remaining two transects were covered during June–July 2025. Each transect was surveyed once during the respective survey period, and all surveys were conducted during daylight hours under suitable field conditions. Transects were walked by an experienced team of three to four observers to cover most of the block, including all elevation ranges and habitat types. Transect locations were not selected randomly but were strategically positioned to provide representative coverage of the major habitat types and elevation gradients, capturing variation in environmental conditions rather than oversampling easily accessible or high-bear-presence areas. Transect routes were arranged in a loop-like form where possible, starting from a central point, progressing toward the block, and ending at the starting point. During the sign survey, observers searched for direct and indirect evidence of Asiatic black bear presence, including direct sightings, pugmarks, claw marks, and scats. For each confirmed record, the date, sign type, elevation, and coordinates (latitude/longitude) were recorded using a Garmin eTrex GPS receiver [47].

Camera trap surveys were conducted during the field survey period from 15 July to 15 August 2025. Thirteen camera trap stations were established across the four surveyed sampling blocks, and one infrared trail camera was deployed at each station. The camera traps consisted of seven ToGuard H100 and six Campark infrared trail cameras. In Block 1, three ToGuard H100 cameras were deployed, and Block 2 was similarly surveyed using three ToGuard H100 cameras. In Block 3, four Campark cameras were deployed, while Block 4 included three camera stations, comprising one ToGuard H100 and three Campark cameras. All cameras were deployed during the same period for 30 consecutive days, resulting in a total effort of 390 camera trap days. Cameras were installed approximately 40–60 cm above ground level on tree trunks and spaced 500–1000 m apart where field conditions allowed. Each camera operated continuously for 24 h per day, recording the date and time for each photograph [48,49,50].

Cameras were placed along trails, stream beds, ridges, and other movement features where bear signs such as pugmarks, scats, or claw marks were observed. Camera locations were strategically selected to maximize the likelihood of detecting Asiatic black bears while ensuring representative coverage of diverse habitat conditions across the four sampling blocks. Placement was guided by field observations of potential movement pathways and signs of bear activity but was not restricted to areas considered highly suitable for bears, allowing sampling across the broader study area. The camera placement conformed to established methodologies employed in wildlife camera trap studies [51,52]. Due to the potential for camera trapping to produce multiple detections of the same individual or at the same location, repeated photographic detections from a single camera station were not regarded as independent occurrence records. Instead, one confirmed photographic record from each camera station was retained as a presence record for habitat modelling. These records were obtained exclusively from the camera traps deployed during the study period.

The sign-based and camera-trap records were combined into a single occurrence dataset after screening (Supplementary Material File S1, Table S1). The photograph in Supplementary Material File S1 (Figure S1) confirmed camera trap detections from the deployed camera stations and was used solely as evidence of species presence. To retain only georeferenced and reliable points, all presence records were checked and screened for missing coordinates, duplicate records, uncertain identifications, and spatial mismatches with the study boundary prior to modelling. In this study, duplicate records were defined as repeated observations from the same geographic location or repeated detections from the same camera trap station. Such records were not treated as independent occurrence points because they could represent repeated observations of the same individual or repeated use of the same location. Therefore, repeated photographic detections from the same camera station were consolidated into a single presence record, while repeated sign observations recorded at identical coordinates were retained only once.

This is important because clustered records can artificially inflate model performance if not handled carefully [8,24]. Because individual identification was not possible from all field signs or camera trap photographs, it is possible that some occurrence records originated from the same individual bear. However, the screening procedures described above, together with balanced spatial cross-validation during model evaluation, were used to reduce the potential influence of repeated or spatially clustered observations.

Explicit spatial thinning was not applied, and therefore no minimum distance threshold was defined; this decision was based on the limited number of available occurrence records and the need to retain sufficient ecological information for HSM. Spatial dependence was considered during model evaluation through balanced spatial cross-validation. This approach provides a more conservative assessment of model transferability compared with random data partitioning.

### 2.3. Environmental Variables

To assess the habitat suitability of the Asiatic black bear, environmental variables were collected from different sources. Climatic variables were obtained from the WorldClim database v2.1 at 30 arc-seconds (ca. 1 km^2^ at the equator; Table 1). The Digital Elevation Model (DEM) was retrieved from the USGS Earth Explorer database and was used to generate the Vector Ruggedness Measure (VRM) using the ‘Vector Ruggedness Measurement’ tool in ArcGIS (Table 1). Land cover data were downloaded from the Esri website (Table 1). Local shapefiles for human settlements were downloaded from the Humanitarian Data Exchange website (Table 1). A shapefile for rivers was obtained from the DIVA-GIS website (Table 1). Distance to human settlements and rivers was calculated by using the ‘Euclidean Distance’ tool in ArcGIS version 10.8.

The environmental predictors used in the HSM were standardised to the same spatial resolution, coordinate system, and extent before analysis. Specifically, the climatic variable (bio19) was obtained at 30 arc-seconds resolution (approximately 1 km^2^), whereas the land cover, settlement, VRM, and water-body layers were resampled and aligned to the same approximately 1 km^2^ spatial resolution. Therefore, all predictor variables and the final HSM grid were based on a consistent spatial resolution of approximately 1 km^2^.

A pairwise Pearson correlation coefficient was calculated among continuous variables, using a conservative threshold of |r| ≥ 0.60 to reduce redundancy among predictors and to identify moderately to strongly correlated variables. This step was included to avoid inflating variable importance, distorting the response curves, and affecting ecological interpretability. This method of reducing the dimensionality of predictor variables prior to modelling is commonly used in SDM studies [24]. The predictors with the highest average correlation were removed when two variables exceeded the correlation threshold.

Finally, five environmental variables were used: precipitation of the coldest quarter (bio19), land cover, settlements, VRM, and rivers (Table 1). These environmental variables were expected to influence Asiatic bear habitat suitability and were chosen because Asiatic black bear occurrence in Himalayan landscapes is commonly associated with forested habitat, rugged terrain, climatic moisture, water availability, and reduced human disturbance [25,29,32,33]. All environmental layers were clipped to the same spatial resolution, coordinate system, and extent (according to the study area boundary) using the Extract by Mask tool in ArcGIS version 10.8.

### 2.4. Habitat Suitability Modelling and Model Validation

The HSM for the Asiatic black bear was developed by using an ensemble modelling method in R version 4.6.1 [42] (https://cran.r-project.org). Ensemble modelling reduces reliance on any single algorithm by integrating predictions from multiple modelling approaches, thereby providing more robust estimates of habitat suitability and helping identify areas where predictions are more consistent across algorithms [43,44]. Six candidate algorithms were selected: Generalized Linear Model (GLM), Generalized Additive Model (GAM), Maxnet, Random Forest (RF), Gradient Boosting Machine (GBM), and Support Vector Machine (SVM). GLM and GAM are regression-based methods; RF and GBM are tree-based machine-learning approaches; SVM is a kernel-based classification method; and Maxnet provides an R-based implementation of the maximum entropy approach. All R-based models were fitted using the same cleaned presence-background dataset and the same predictors retained after collinearity screening. Detailed and commented R codes and packages used in this study were included in Supplementary Material File S2.

Model performance was evaluated using balanced spatial cross-validation. Spatial validation was implemented because occurrence records collected from field surveys and camera trapping may show geographic clustering, and random train-test splitting can overestimate accuracy when training and testing locations are geographically close. Presence records were partitioned into spatial clusters using k-means clustering based on geographic coordinates. The initial target was five spatial folds; however, the number of folds was reduced to four because each fold was required to contain a minimum of 20 presence records to ensure reliable calculation of evaluation metrics. Background points were assigned to the nearest presence-fold centroid so that each fold contained both presence and background data. The final four-fold spatial validation design provided spatial separation between training and testing datasets and resulted in a more conservative estimate of model transferability than random validation. Model performance was assessed using the Area Under the Curve (AUC), the True Skill Statistic (TSS), PR-AUC, and the Boyce index. Performance metrics were computed separately for each spatial fold and summarised as mean ± standard deviation. High variation among folds was interpreted as evidence of spatial heterogeneity in model transferability. After model evaluation, candidate models were screened for inclusion in the ensemble. The preferred rule was to include only models with AUC ≥ 0.80 and TSS ≥ 0.60. Because no model met both thresholds in balanced spatial cross-validation, a transparent fallback rule was applied: the top three models, ranked by mean AUC, were selected. The final ensemble included Maxnet, GLM, and SVM. The ensemble suitability map was treated as the primary ecological output because it retains the full suitability gradient and avoids information loss from thresholding. The ensemble suitability map was reclassified into a binary suitable/unsuitable map using a weighted TSS threshold. Habitat area was calculated by summing the area of raster cells in each suitability class:

A_C_ = ∑a_i_

where A_c_ is the area of the habitat class c, and a_i_ is the area of each raster cell in km^2^.

## 3. Results

### 3.1. Occurrence Records Used for Modelling Analysis

**Figure 2:**
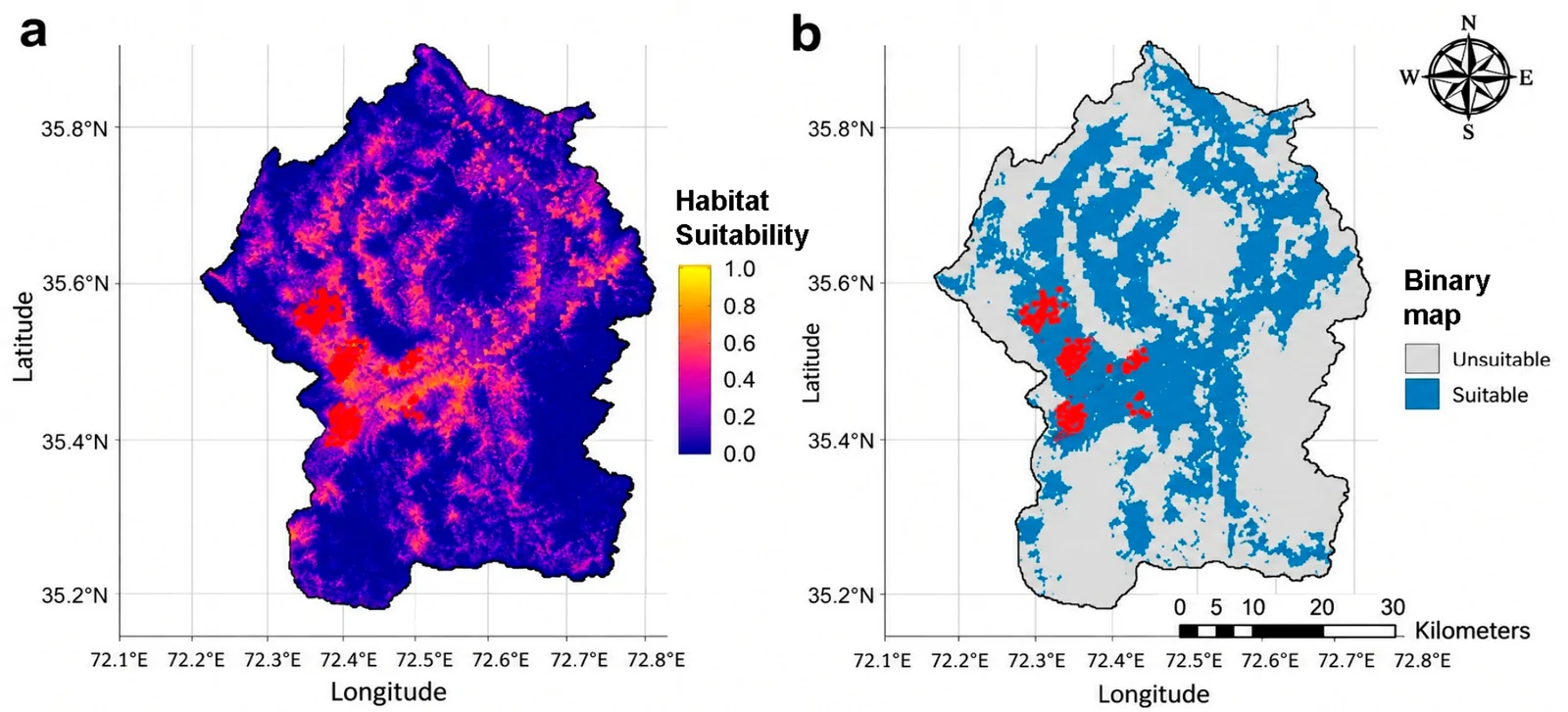
Habitat suitability modelling maps of the Asiatic black bear: (**a**) continuous; and (**b**) binary presence/absence. Red points show Asiatic black bear presence records.

After removing duplicate, uncertain, and spatially unreliable records, field surveys and camera trapping yielded 126 confirmed records of Asiatic black bear presence (Supplementary Material File S1, Table S1 and Figure 2). These records comprised both direct and indirect field signs, as well as independent camera-trap detections (Supplementary Material File S1, Figure S1). Most presence records were located within or close to areas of moderate to high habitat suitability, indicating a strong spatial agreement between model predictions and field observations (Figure 2a,b).

### 3.2. Habitat Suitability of the Asiatic Black Bear

The HSM output indicated relatively higher suitability in north-western and central portions of the study area (Figure 2a,b).

The binary map, created by using the weighted TSS threshold, showed that 1061.22 km^2^ of the study area was classified as suitable, mainly included between 400 and 3500 m a.s.l. (Figure 2b).

### 3.3. Variable Importance

**Figure 3:**
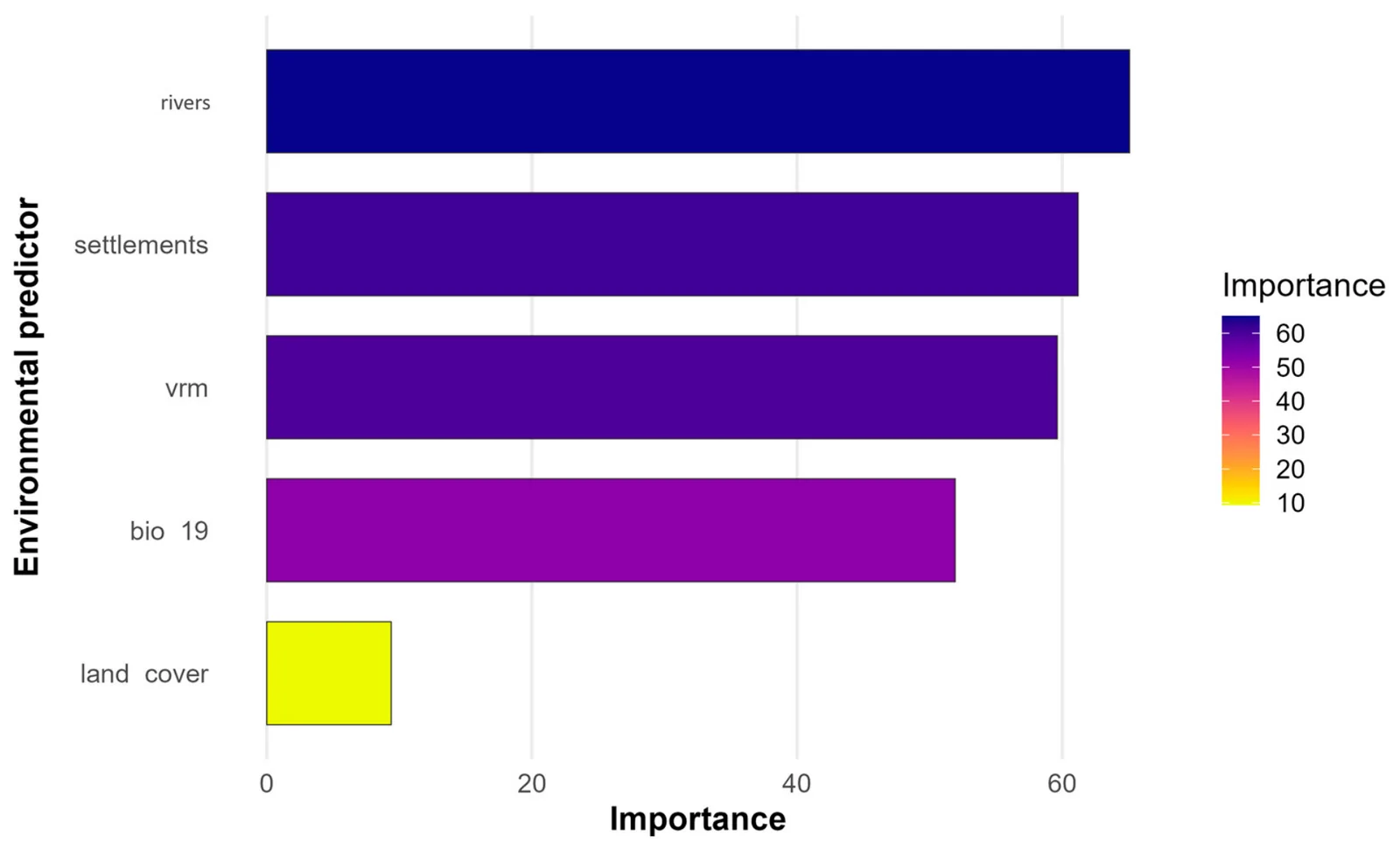
Habitat suitability modelling variable importance. Higher values indicate greater contribution to node purity and model discrimination. vrm = vector ruggedness measures; and bio 19 = Precipitation of the coldest quarter.

HSM variable importance ranked distance to rivers as the most influential predictor, followed by the distance to settlements, VRM, bio19, and land cover (Figure 3). The spatial patterns in the habitat suitability maps reflect the heterogeneous distribution of environmental conditions throughout the mountainous terrain. Areas with high suitability were concentrated in the northern and north-central portions of the study area, consistent with denser river networks and greater distance from settlements (Figure 3).

### 3.4. Model Validation

Comparative modelling was assessed using a balanced spatial cross-validation approach. Balanced spatial validation demonstrated moderate performance across the evaluated algorithms. Maxnet emerged as the best-performing model, with a mean AUC of 0.784 ± 0.188 and a mean TSS of 0.532 ± 0.336. GLM showed comparable performance in terms of TSS (0.526 ± 0.290) but yielded a lower mean AUC (0.739 ± 0.189). Other models, including SVM, GAM, GBM, and RF, demonstrated either reduced or more variable performance. All models showed substantial spatial variability, suggesting that predictive performance varied considerably across spatial folds (Table 2).

As no candidate model met the preferred ensemble criteria (AUC ≥ 0.80 and TSS ≥ 0.60), the ensemble model was assembled using the fallback procedure, which selected the three highest-ranked models based on mean AUC. The final ensemble comprised Maxnet, GLM, and SVM, with AUC-derived weights of 0.347, 0.327, and 0.326, respectively.

## 4. Discussion

The current study provides a field-based sign survey, camera trap detection, and model-supported habitat suitability assessment of the Asiatic black bear in the Kalam Valley in northern Pakistan.

### 4.1. Habitat Suitability and Model Validation

Our study found that high habitat suitability values for the Asiatic black bear are concentrated in the north-western and central parts of the study area [17,23]. This pattern suggested that mountainous, rugged, moist, and low-disturbed areas in the modelling domain were the most suitable [17]. On the other hand, the remaining portions of the Kalam Valley were consistently classified as less suitable, mainly due to very high elevation (e.g., >4000 m a.s.l.).

The final ensemble model included Maxnet, GLM, and SVM as the three most reliable models based on mean AUC. This model-selection approach was chosen because none of the models met the stricter criteria of AUC ≥ 0.80 and TSS ≥ 0.60 during balanced spatial cross-validation. As a result, the ensemble should be seen as a cautious estimate of relative suitability rather than an exact prediction of occurrence. This perspective aligns with the presence-background study design and follows standard recommendations, which suggest that SDM outputs indicate relative environmental suitability or favourability rather than a confirmed likelihood of occupancy, especially when detection or non-detection data are unavailable [39,42].

The ensemble model identified approximately 1061.22 km^2^ as a suitable zone. The concentration of suitable habitat in the core of the Kalam Valley makes ecological sense [9,17]. These areas are characterised by rugged terrain, forested slopes, and hydrological features, along with environmental conditions like those in locations where Asiatic black bears have been confirmed. Asiatic black bears are typically found in forested mountain areas, in our case from 400 to 3500 m a.s.l., that offer food, shelter, denning spots, and protection from human activity [8,24,29]. The predicted pattern thereby supports the ecological expectation that suitable Asiatic black bear habitats in the study area are not evenly distributed but are instead concentrated in the more favourable mountain zones [23,25,27]. Although approximately 1061 km^2^ was identified as suitable habitat for the Asiatic black bear, the present study does not allow estimation of the number of individuals that this area could support. Habitat suitability models identify areas with environmental conditions favourable for species occurrence but do not directly provide information on population density or carrying capacity. Estimating the number of individuals supported by the predicted suitable habitat would require additional information, including population density estimates, home-range size, demographic parameters, habitat-specific resource availability, and carrying capacity assessments. Therefore, the predicted suitable area should be interpreted as an indicator of potential suitability areas rather than an estimate of population size.

The low or null suitability values observed in other parts of the study area were mainly attributable to greater human presence compared with suitable areas, as well as to the occurrence of high-altitude terrain. Human activities, habitat loss, and landscape fragmentation negatively affect the survival of Asiatic black bears, particularly in human-dominated landscapes [12,23,25,32].

Our results indicate that habitat suitability for the Asiatic black bear is linked to the presence of rivers. It is well known that, in general, vegetation productivity, foraging opportunities, movement routes, and habitat use in mountain valleys are associated with the presence of water. Therefore, the importance of water-related variables is consistent with the ecology of a wide-ranging omnivore that uses riparian and forested ecosystems for movement and access to resources. Ecologically, VRM is important, as rugged terrain can provide shelter, denning opportunity, escape cover, and less accessibility to human-dominated landscapes. In a mountainous landscape where human influence is at its maximum, rugged areas may function as refuges where large mammals can reduce their exposure to disturbance. Similar associations between terrain complexity and habitat suitability have been documented in studies of the Asiatic black bear and other large mammals in the Himalayan mountain ecosystem [32,33]. Precipitation of the coldest period (bio19) was another important predictor, showing that this variable may capture broader environmental conditions related to winter severity, snowfall, vegetation structure, and seasonal resource availability. Previous studies in Pakistan and the Himalayas, based on habitat suitability assessments of the Asiatic black bear, identified climatic variables as important drivers of its distribution [22,29]. Land cover was less important in our results, but this does not mean it is ecologically unimportant. Across much of its range, the Asiatic black bear is strongly associated with forested habitats. In fact, the influence of forest and vegetation structure on the Asiatic black bear habitat suitability has been repeatedly shown in previous studies [8,24,29].

### 4.2. Conservation Implications

Our findings identified the Kalam Valley as a priority habitat for the conservation of the Asiatic black bear. These areas should be protected and monitored to support targeted conservation, disturbance reduction, and conflict-prevention planning. The habitat suitability assessment conducted in this study was specifically developed for the upper reaches of Kalam Valley within the Kalam–Swat region and should therefore be interpreted within this modelling domain. While other areas potentially suitable for Asiatic black bears may exist outside the current study area, identifying these locations would necessitate additional occurrence data and HSM at a larger spatial scale to improve conservation strategy for this apical predator [9,17].

The significance of rivers and rugged terrain indicates that riparian forest zones, stream corridors, and rugged forested slopes might be especially vital for Asiatic black bear conservation. These habitats are likely to offer movement pathways, cover, feeding resources, and refuge from human disturbance. Therefore, management strategies should emphasize the preservation of forested watercourses, implement measures to prevent habitat degradation near streams, and minimize disturbances in rugged forested regions.

Beyond habitat availability, sustaining connectivity among suitable habitat patches is vital for long-term conservation objectives. In mountainous regions such as Kalam Valley, habitat fragmentation resulting from expanding settlements, road development, agricultural activities, tourism infrastructure, and other anthropogenic disturbances can hinder bear movement and diminish access to otherwise suitable habitats. Consequently, conservation strategies should prioritise not only the protection of main core areas but also the identification and preservation of movement corridors, as well as the mitigation of barriers that impede landscape connectivity.

Settlement-related disturbance is also an important consideration for the conservation of the Asiatic black bear. The risk of human–bear interactions may increase where high-suitability habitat overlaps with settlements, tourism routes, grazing areas, or forest resource extraction zones. In another part of northern Pakistan, a similar concern was reported, linking Asiatic black bear conflict to spatial overlap between human land use and bear habitat [2,23]. Therefore, conservation planning should combine habitat protection with community-based conflict mitigation, such as awareness programs, waste management around tourism areas, livestock protection, and local reporting systems for bear presence and conflict events [9,17].

### 4.3. Limitations and Future Research

This study has a few limitations worth keeping in mind when interpreting the results. First, the models used presence-background data instead of systematic detection or non-detection data. While this approach is well suited to elusive species in rugged terrain, it means that the results reflect relative habitat suitability rather than the actual probability of occupancy [39,42]. In the future, repeated camera-trap surveys could help to more directly estimate detection probability and occupancy. Although field data were collected between May 2024 and September 2025, seasonal HSM was not conducted because the available occurrence records were insufficient for developing reliable season-specific models. Dividing the available presence records into different seasons would substantially reduce the sample size and may result in unstable model predictions. Furthermore, the objective of the present study was to identify overall habitat suitability patterns and the major environmental factors influencing Asiatic black bear distribution in the study area. Future studies involving larger datasets, repeated seasonal surveys, and balanced occurrence records across seasons are recommended to investigate seasonal variation in habitat use and habitat suitability.

Second, to improve future studies, it would be helpful to expand systematic sampling to currently under-surveyed areas, especially those with high suitability or high uncertainty. This would provide a more complete and reliable understanding of the species’ distribution.

Third, the environmental predictors used in this study covered a wide range of climatic, topographic, hydrological, land-cover, and settlement factors, giving us a broad overview. However, they did not directly capture important factors such as seasonal food availability, hunting pressure, road traffic, livestock density, or tourism intensity. These factors can significantly influence how Asiatic black bears use their habitat at finer spatial and seasonal scales. Incorporating these variables in future studies could make ecological insights more meaningful for conservation management.

Fourth, this research did not explicitly incorporate habitat connectivity modelling. The concentration of highly suitable habitats in the northern and north-central regions of Swat District indicates that these zones may serve as core habitats; however, the pathways facilitating movement corridors and potential barriers remain unidentified. Future investigations should integrate habitat suitability assessments with least-cost path modelling, circuit theory, or graph-based connectivity methods to delineate potential corridors and barriers. This approach is particularly pertinent to Kalam Valley, where tourism development, infrastructure expansion, and settlement growth threaten to increasingly fragment the habitat.

We recommended that future research investigate seasonal habitat modelling. Asiatic black bears are expected to modify their habitat utilization throughout the year, influenced by factors such as snow cover, food availability, denning requirements, and human activities. The development of seasonal models could facilitate the identification of specific summer foraging sites, autumn feeding locations, winter denning areas, and potential conflict zones. Such an approach would provide a more accurate ecological framework for guiding targeted conservation efforts within the Kalam Valley.

## 5. Conclusions

The current study identified the habitat suitability for the Asiatic black bear in the Kalam Valley. Our HSM results consistently indicated that higher relative habitat suitability is concentrated in the north-western and central parts of the study area. Terrain ruggedness, water availability, climatic moisture, and settlement-related disturbance emerged as important predictors, indicating that both natural environmental gradients and human activities impact habitat suitability.

Our results provide practical guidance for conservation planning in the study area. Riparian forests, rugged slopes, and forested transition zones require careful attention because they may provide movement corridors, shelter, food, and refuge habitats. Areas where suitable habitats overlap with settlements, tourism routes, grazing lands, or forest resource use should be managed through conflict-sensitive conservation strategies, including community awareness programs, waste management policies, livestock protection measures, and local monitoring of bear activity.

Although the models provide useful spatial guidance, the outputs should be interpreted as relative habitat suitability rather than confirmed occupancy probability. Future research should expand systematic camera-trap surveys, examine seasonal habitat use, integrate fine-scale disturbance and food-resource variables, and evaluate habitat connectivity among suitable patches. Overall, this study provides a reproducible framework for identifying priority Asiatic black bear habitats in northern Pakistan and supports evidence-based conservation planning for a vulnerable species in a rapidly changing mountain landscape.

## Figures and Tables

**Table 1:** Environmental predictors used in the Asiatic black bear models.

Variable Codes	Description	Download Link	Unit
bio19	Precipitation of the coldest quarter (Continuous variable)	https://www.worldclim.org/data/worldclim21.html accessed on 27 September 2026	mm
Land cover	Forest, grassland, cropland, built-up area, and barren land (Categorical variable)	https://livingatlas.arcgis.com/landcover/ accessed on 27 September 2026	Class code
Settlements	Distance/proximity to human settlements (Continuous variable)	https://data.humdata.org accessed on 27 September 2026	km
VRM	Vector ruggedness measures (Continuous variable)	https://earthexplorer.usgs.gov/ accessed on 27 September 2026	Unitless index
River	Distance/proximity to river (Continuous variable)	https://www.diva-gis.org/data accessed on 27 September 2026	km

**Table 2:** Balanced spatial cross-validation performance of candidate SDM algorithms. Mean and standard deviation of AUC, TSS, PR-AUC, and Boyce index across balanced spatial validation folds. Acronyms as follows: Generalized Linear Model (GLM), Generalized Additive Model (GAM), Maxnet, Random Forest (RF), Gradient Boosting Machine (GBM), and Support Vector Machine (SVM).

Model	AUC Mean ± SD	TSS Mean ± SD	PR-AUC Mean	Boyce Mean
Maxnet	0.78 ± 0.18	0.53 ± 0.33	0.11	0.64
GLM	0.73 ± 0.18	0.52 ± 0.29	0.14	0.46
SVM	0.73 ± 0.17	0.47 ± 0.22	0.10	0.48
GAM	0.73 ± 0.17	0.48 ± 0.30	0.18	0.56
GBM	0.72 ± 0.20	0.43 ± 0.31	0.09	0.49

## Data Availability

All the data used in this study are available in the main text and Supplementary Materials.

## Supplementary Materials

The following supporting information can be downloaded at: https://www.mdpi.com/article/10.3390/ani16193080/s1. File S1: Table S1. Presence records of the Asiatic black bear (*Ursus thibetanus*) used in the species distribution models; Figure S1. Picture by camera trapping of the Asiatic black bear (*Ursus thibetanus*) in the Kalam–Swat landscape, Pakistan; File S2: R codes used for the Asiatic black bear habitat suitability.
