# Supplementary material for: Mapping Habitat Suitability of the Asiatic Black Bear (Ursus thibetanus) in the Kalam Valley, Pakistan: An Ensemble Modelling Approach
Source: Animals (Basel). 2026 Sep 30;16(19):3080. doi: 10.3390/ani16193080 (PMC13663381; doi:10.3390/ani16193080)
Supplement: Supplementary file 1 [file animals-16-03080-s001.zip › Supplementary Material File S1.pdf]

**Supplementary material 1**

**Table S1.** Presence records of the Asiatic black bear (*Ursus thibetanus*) used in the species distribution models.

| Number | Location | Latitude | Longitude | Elevation | Sign Type |
|---|---|---|---|---|---|
| 1 | Gous | 35.31348 | 72.29470 | 3,185 | Scat |
| 2 | Gous | 35.31342 | 72.29333 | 3,244 | Scat |
| 3 | Gous | 35.31362 | 72.29341 | 3,244 | Scat |
| 4 | Gous | 35.31367 | 72.29475 | 3,215 | Scat |
| 5 | Desan | 35.28544 | 72.29702 | 3,026 | Scat |
| 6 | Gous | 35.30304 | 72.28584 | 2,684 | Scat |
| 7 | Gous | 35.30966 | 72.29366 | 2,929 | Scat |
| 8 | Gous | 35.30304 | 72.28503 | 2,885 | Scat |
| 9 | Gous | 35.30956 | 72.29368 | 2,928 | Scat |
| 10 | Gous | 35.31367 | 72.29447 | 3,215 | Scat |
| 11 | Gous | 35.30474 | 72.28715 | 2,783 | Scat |
| 12 | Gous | 35.30789 | 72.29356 | 2,938 | Scat |
| 13 | Gous | 35.30520 | 72.29194 | 2,751 | Scat |
| 14 | Gous | 53.31044 | 72.29304 | 2,993 | Scat |
| 15 | Gous | 35.30805 | 72.28470 | 3,074 | Scat |
| 16 | Gous | 35.30998 | 72.29352 | 2,944 | Scat |
| 17 | Gous | 35.30786 | 72.28531 | 3,051 | Scat |
| 18 | Gous | 35.31295 | 72.28959 | 3,262 | Scat |
| 19 | Gous | 35.30609 | 72.28756 | 2,858 | Scat |
| 20 | Desan | 35.28686 | 72.29651 | 3,007 | Scat |
| 21 | Desan | 35.28608 | 72.29574 | 3,087 | Scat |
| 22 | Desan | 35.28418 | 72.29406 | 3,130 | Scat |
| 23 | Kekijdan | 35.30652 | 72.27337 | 3,137 | Photograph |
| 24 | Kekijdan | 35.30512 | 72.27281 | 3,110 | Photograph |
| 25 | Gous | 35.30464 | 72.28479 | 3,035 | Scrape |
| 26 | Gous | 35.30835 | 72.28483 | 3,096 | Scrape |
| 27 | Gous | 35.31213 | 72.29361 | 3,093 | Pugmark |
| 28 | Desan | 35.28611 | 72.29623 | 3,039 | Pugmark |
| 29 | Shahi Bagh | 35.58991 | 72.37296 | 4,354 | Digging |
| 30 | Dash-E-Laila | 35.44602 | 72.40614 | 3,369 | Scrap |
| 31 | Palasher Banda | 35.54138 | 72.39836 | 3,187 | Digging |
| 32 | Palasher Banda | 35.52786 | 72.41976 | 2,451 | Scrape |
| 33 | Dash-E-Laila | 35.45168 | 72.41298 | 3,234 | Scrape |
| 34 | Shahi Bagh | 35.57516 | 72.36677 | 4,025 | Digging |
| 35 | Shahi Bagh | 35.59467 | 72.36677 | 4,597 | Tree scrap |
| 36 | Shahi Bagh | 35.59277 | 72.37914 | 4,108 | Digging |
| 37 | Dash-E-Laila | 35.42671 | 72.37976 | 3,229 | Tree scrap |
| 38 | Shahi Bagh | 35.58658 | 72.38105 | 4,005 | Scrape |
| 39 | Dash-E-Laila | 35.46119 | 72.39127 | 3,329 | Pugmark |
| 40 | Dash-E-Laila | 35.46209 | 72.41388 | 2,842 | Pugmark |
| 41 | Dash-E-Laila | 35.43472 | 72.39811 | 3,556 | Tree scrap |
| 42 | Shahi Bagh | 35.56945 | 72.36391 | 3,661 | Scrape |

| | | | | | |
|---|---|---|---|---|---|
| 43 | Palasher Banda | 35.53284 | 72.42576 | 2,861 | Digging |
| 44 | Palasher Banda | 35.52428 | 72.40577 | 2,501 | Tree scrap |
| 45 | Palasher Banda | 35.53046 | 72.40958 | 2,436 | Pugmark |
| 46 | Palasher Banda | 35.52142 | 72.39768 | 2,771 | Pugmark |
| 47 | Palasher Banda | 35.50572 | 72.39959 | 3,311 | Scrape |
| 48 | Dash-E-Laila | 35.43294 | 72.40882 | 3,929 | Tree scrap |
| 49 | Dash-E-Laila | 35.45852 | 72.41001 | 2,993 | Digging |
| 50 | Shahi Bagh | 35.57564 | 72.37581 | 4,443 | Pugmark |
| 51 | Shahi Bagh | 35.57897 | 72.38247 | 4,193 | Digging |
| 52 | Shahi Bagh | 35.56469 | 72.36154 | 3,461 | Scrape |
| 53 | Dash-E-Laila | 35.44483 | 72.39752 | 3,371 | Tree scrap |
| 54 | Palasher Banda | 35.51954 | 72.40822 | 2,508 | Pugmark |
| 55 | Palasher Banda | 35.53391 | 72.4053 | 2,879 | Digging |
| 56 | Dash-E-Laila | 35.45584 | 72.39663 | 2,899 | Tree scrap |
| 57 | Shahi Bagh | 35.56469 | 72.35392 | 3,185 | Scrape |
| 58 | Shahi Bagh | 35.56755 | 72.34678 | 3,048 | Tree scrap |
| 59 | Shahi Bagh | 35.57135 | 72.35345 | 3,544 | Pugmark |
| 60 | Dash-E-Laila | 35.44924 | 72.39666 | 3,231 | Digging |
| 61 | Dash-E-Laila | 35.44989 | 72.40525 | 3,307 | Scrape |
| 62 | Dash-E-Laila | 35.45554 | 72.40436 | 3,101 | Pugmark |
| 63 | Shahi Bagh | 35.60038 | 72.36487 | 4,363 | Pugmark |
| 64 | Dash-E-Laila | 35.45911 | 72.40495 | 2,949 | Pugmark |
| 65 | Palasher Banda | 35.50715 | 72.41047 | 2,947 | Pugmark |
| 66 | Palasher Banda | 35.5263 | 72.40102 | 2,575 | Digging |
| 67 | Dash-E-Laila | 35.42639 | 72.39068 | 3,498 | Digging |
| 68 | Shahi Bagh | 35.58896 | 72.36439 | 4,655 | Pugmark |
| 69 | Shahi Bagh | 35.56993 | 72.38438 | 3,818 | Scrape |
| 70 | Shahi Bagh | 35.5585 | 72.35725 | 2,952 | Digging |
| 71 | Dash-E-Laila | 35.45713 | 72.38765 | 3,177 | Pugmark |
| 72 | Shahi Bagh | 35.57516 | 72.36677 | 4,025 | Tree scrape |
| 73 | Dash-E-Laila | 35.44067 | 72.38978 | 3,211 | Tree scrape |
| 74 | Shahi Bagh | 35.59467 | 72.36677 | 4,597 | Digging |
| 75 | Palasher Banda | 35.51286 | 72.38864 | 3,197 | Tree scrape |
| 76 | Palasher Banda | 35.52786 | 72.41976 | 2,451 | Digging |
| 77 | Palasher Banda | 35.51095 | 72.40673 | 2,926 | Pugmark |
| 78 | Palasher Banda | 35.49858 | 72.39768 | 3,454 | Pugmark |
| 79 | Palasher Banda | 35.50715 | 72.39245 | 3,645 | Digging |
| 80 | Shahi Bagh | 35.56897 | 72.37772 | 4,198 | Scrape |
| 81 | Shahi Bagh | 35.58896 | 72.36439 | 4,655 | Scrape |
| 82 | Palasher Banda | 35.52786 | 72.41976 | 2,451 | Pugmark |
| 83 | Palasher Banda | 35.54138 | 72.42539 | 3,041 | Digging |
| 84 | Dash-E-Laila | 35.44959 | 72.3883 | 3,100 | Tree scrape |
| 85 | Dash-E-Laila | 35.44989 | 72.40942 | 3,300 | Pugmark |
| 86 | Shahi Bagh | 35.58849 | 72.35511 | 4,242 | Scrape |
| 87 | Dash-E-Laila | 35.43294 | 72.40138 | 3,665 | Digging |
| 88 | Palasher Banda | 35.52333 | 72.39197 | 2,807 | Tree scrape |
| 89 | Palasher Banda | 35.51143 | 72.39959 | 3,293 | Pugmark |

| | | | | | |
|---|---|---|---|---|---|
| 90 | Dash-E-Laila | 35.44156 | 72.40376 | 3,542 | Pugmark |
| 91 | Shahi Bagh | 35.57992 | 72.36201 | 4,155 | Scrape |
| 92 | Shahi Bagh | 35.60419 | 72.38771 | 3,813 | Digging |
| 93 | Shahi Bagh | 35.58706 | 72.39437 | 3,410 | Tree scrape |
| 94 | Palasher Banda | 35.54093 | 72.41149 | 2,480 | Scrape |
| 95 | Palasher Banda | 35.50143 | 72.40387 | 3,264 | Digging |
| 96 | Shahi Bagh | 35.57849 | 72.33869 | 3,719 | Pugmark |
| 97 | Palasher Banda | 35.51809 | 72.40149 | 2,874 | Pugmark |
| 98 | Palasher Banda | 35.51571 | 72.39388 | 3,188 | Scrape |
| 99 | Shahi Bagh | 35.58896 | 72.36439 | 4,655 | Pugmark |
| 100 | Palasher Banda | 35.51333 | 72.41291 | 2,625 | Scrape |
| 101 | Palasher Banda | 35.5219 | 72.41482 | 2,344 | Tree scrape |
| 102 | Shahi Bagh | 35.58325 | 72.36772 | 4,575 | Pugmark |
| 103 | Desan | 35.45632 | 72.48449 | 3,478 | Pugmark |
| 104 | Desan | 35.45721 | 72.49549 | 3,571 | Pugmark |
| 105 | Shahi Bagh | 35.58373 | 72.3444 | 4,189 | Tree scrape |
| 106 | Palasher Banda | 35.53189 | 72.39959 | 3,041 | Digging |
| 107 | Palasher Banda | 35.53332 | 72.41577 | 2,401 | Scrape |
| 108 | Gous | 35.52038 | 72.50068 | 3,045 | Pugmark |
| 109 | Dash-E-Laila | 35.44305 | 72.41477 | 3,526 | Scrape |
| 110 | Dash-E-Laila | 35.43978 | 72.39395 | 3,337 | Pugmark |
| 111 | Gous | 35.53109 | 72.498 | 3,791 | Digging |
| 112 | Shahi Bagh | 35.56754 | 72.34044 | 2,929 | Digging |
| 113 | Desan | 35.46458 | 72.47953 | 3,246 | Digging |
| 114 | Desan | 35.45332 | 72.50319 | 3,623 | Scrape |
| 115 | Shahi Bagh | 35.58325 | 72.36772 | 4,575 | Digging |
| 116 | Dash-E-Laila | 35.45078 | 72.41774 | 3,215 | Pugmark |
| 117 | Palasher Banda | 35.51809 | 72.4191 | 2,321 | Tree scrape |
| 118 | Palasher Banda | 35.53998 | 72.41624 | 2,375 | Scrape |
| 119 | Dash-E-Laila | 35.46536 | 72.39692 | 3,535 | Tree scrape |
| 120 | Shahi Bagh | 35.57421 | 72.34678 | 3,480 | Digging |
| 121 | Shahi Bagh | 35.57469 | 72.35868 | 3,861 | Tree scrape |
| 122 | Dash-E-Laila | 35.43685 | 72.41356 | 3,752 | Pugmark |
| 123 | Palasher Banda | 35.51809 | 72.38674 | 2,851 | Scrape |
| 124 | Palasher Banda | 35.53046 | 72.39483 | 2,808 | Tree scrape |
| 125 | Gous | 35.52008 | 72.49354 | 2,964 | Pugmark |
| 126 | Gous | 35.52811 | 72.48759 | 3,830 | Scrape |

**Figure**

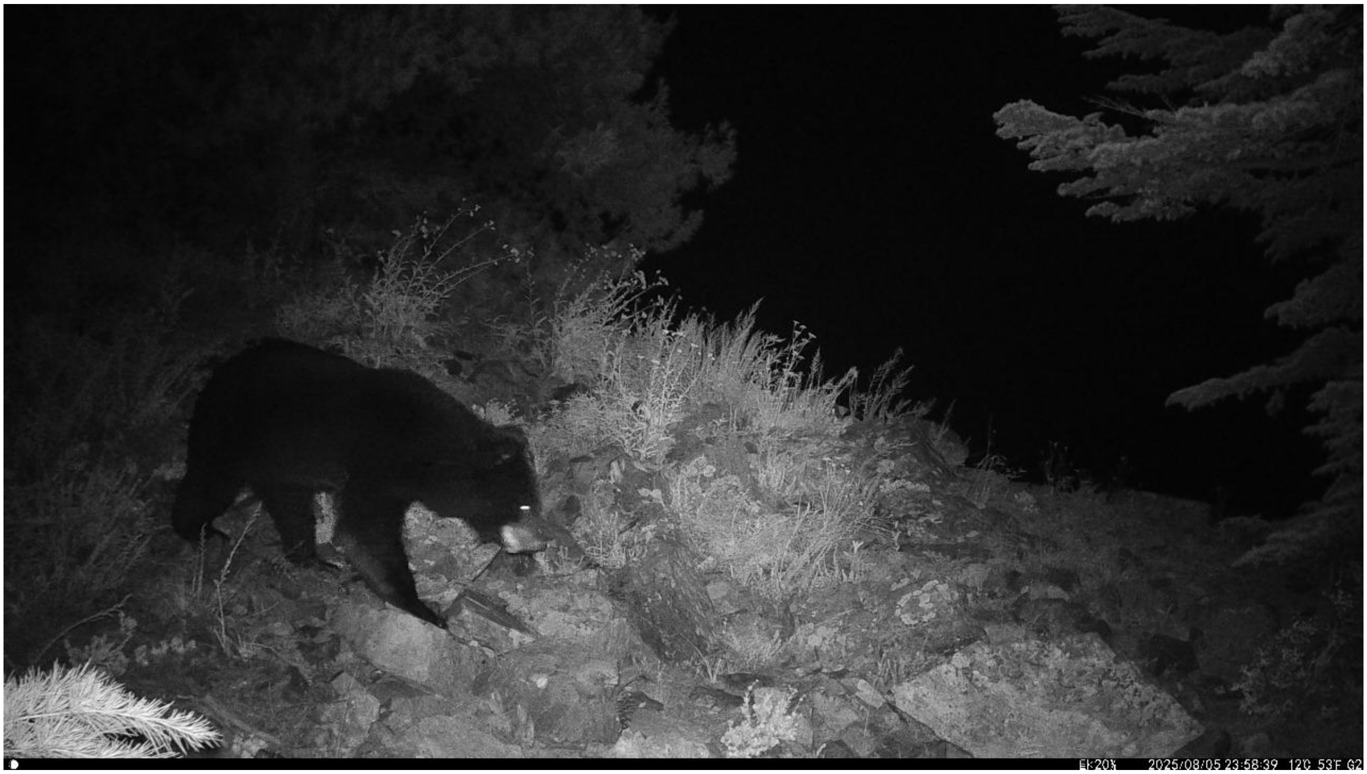


**Figure S1.** Picture by camera trapping of the Asiatic black bear (*Ursus thibetanus*) in the Kalam valley, Pakistan.
