# Supplementary material for: Mapping Habitat Suitability of the Asiatic Black Bear (Ursus thibetanus) in the Kalam Valley, Pakistan: An Ensemble Modelling Approach
Source: Animals (Basel). 2026 Sep 30;16(19):3080. doi: 10.3390/ani16193080 (PMC13663381; doi:10.3390/ani16193080)
Supplement: Supplementary file 1 [file animals-16-03080-s001.zip › Supplementary Material File S2.pdf]

Supplementary material 2

R codes used for the Asiatic black bear habitat suitability

```r
# Main fix:

# - Replaces failed 10-block validation that produced only 2 usable folds.

# - Uses balanced spatial k-means folds based on presence locations.

# - Ensures each fold has enough presences for defensible spatial validation.

#

# Outputs:

# outputs/tables/

# outputs/maps/

# outputs/figures/

# outputs/logs/

############################################################


rm(list = ls())

gc()


options(stringsAsFactors = FALSE)

options(warn = 1)


############################################################

# 0. USER SETTINGS

############################################################


K_FOLDS_TARGET <- 5

MIN_PRESENCES_PER_FOLD <- 15


COLLINEARITY_CUTOFF <- 0.60
```

```r
ENSEMBLE_MIN_AUC <- 0.80
ENSEMBLE_MIN_TSS <- 0.60
ENSEMBLE_TOP_N_FALLBACK <- 3

GBM_TREES <- 1000
RF_TREES <- 500
RUN_SVM <- TRUE

LOW_MOD_Q <- 0.50
MOD_HIGH_Q <- 0.80

# Optional: keep FALSE if you want to use the full Swat-wide background.
# Set TRUE only if reviewer asks for accessible-area/background correction.
USE_ACCESSIBLE_AREA_BACKGROUND <- FALSE
ACCESSIBLE_BUFFER_KM <- 30
TARGET_BACKGROUND_N <- 10000

############################################################
# 1. PACKAGES
############################################################

packages <- c(
  "terra", "sf", "readr", "dplyr", "tidyr",
  "pROC", "PRROC", "randomForest", "gbm",
  "mgcv", "e1071", "maxnet", "ggplot2",
  "viridis", "scales"
)
```

```
to_install <- packages[!(packages %in% rownames(installed.packages()))]

if (length(to_install) > 0) {
  install.packages(to_install, dependencies = TRUE)
}

suppressPackageStartupMessages({
  library(terra)
  library(sf)
  library(readr)
  library(dplyr)
  library(tidyr)
  library(pROC)
  library(PRROC)
  library(randomForest)
  library(gbm)
  library(mgcv)
  library(e1071)
  library(maxnet)
  library(ggplot2)
  library(viridis)
  library(scales)
})

##################################################################
# 2. HELPER FUNCTIONS
##################################################################
```

```r
msg <- function(...) cat("\n", paste0(...), "\n", sep = "")

fail <- function(...) stop(paste0(...), call. = FALSE)

safe_dir_create <- function(x) {
  if (!dir.exists(x)) dir.create(x, recursive = TRUE, showWarnings = FALSE)
}

make_valid_names <- function(x) {
  x <- trimws(x)
  x <- gsub("\\s+", "_", x)
  x <- gsub("[^A-Za-z0-9_]", "_", x)
  x <- gsub("_+", "_", x)
  x
}

get_folder <- function() {
  d <- NA_character_

  if (.Platform$OS.type == "windows") {
    d <- tryCatch(
      choose.dir(caption = "Select folder containing Swat-wide black bear files"),
      error = function(e) NA_character_
    )
  }

  if (is.na(d) || !nzchar(d)) {
```

```
    msg("Folder picker unavailable. Paste the full folder path.")
    d <- readline("Folder path: ")
  }

  d <- normalizePath(d, winslash = "/", mustWork = FALSE)

  if (!dir.exists(d)) fail("Folder does not exist: ", d)

  d
}

find_one <- function(files, pattern, label, required = TRUE) {
  hit <- files[grepl(pattern, basename(files), ignore.case = TRUE)]
  hit <- hit[!grepl("\\.ovr$|\\.xml$|\\.aux\\.xml$", hit, ignore.case = TRUE)]

  if (length(hit) == 0) {
    if (required) fail("Could not find ", label, ". Pattern used: ", pattern)
    return(NA_character_)
  }

  hit[1]
}

find_col <- function(df, candidates, label) {
  nms <- names(df)
  clean <- tolower(gsub("[^a-z0-9]", "", nms))
  cand <- tolower(gsub("[^a-z0-9]", "", candidates))
  idx <- match(cand, clean)
```

```r
  idx <- idx[!is.na(idx)]

  if (length(idx) == 0) {
    fail(
      "Cannot find required column for ", label,
      ". Available columns: ", paste(nms, collapse = ", ")
    )
  }

  nms[idx[1]]
}

calc_auc <- function(obs, pred) {
  obs <- as.numeric(obs)
  pred <- as.numeric(pred)

  ok <- complete.cases(obs, pred)
  obs <- obs[ok]
  pred <- pred[ok]

  if (length(unique(obs)) < 2) return(NA_real_)

  roc_obj <- pROC::roc(obs, pred, quiet = TRUE)
  as.numeric(pROC::auc(roc_obj))
}

calc_pr_auc <- function(obs, pred) {
  obs <- as.numeric(obs)
```

```
  pred <- as.numeric(pred)

  ok <- complete.cases(obs, pred)
  obs <- obs[ok]
  pred <- pred[ok]

  if (length(unique(obs)) < 2) return(NA_real_)

  pr <- tryCatch(
    PRROC::pr.curve(
      scores.class0 = pred[obs == 1],
      scores.class1 = pred[obs == 0],
      curve = FALSE
    ),
    error = function(e) NULL
  )

  if (is.null(pr)) return(NA_real_)

  pr$auc.integral
}

calc_tss <- function(obs, pred) {
  obs <- as.numeric(obs)
  pred <- as.numeric(pred)

  ok <- complete.cases(obs, pred)
  obs <- obs[ok]
```

```
pred <- pred[ok]

if (length(unique(obs)) < 2) {
  return(data.frame(
    TSS = NA_real_,
    threshold = NA_real_,
    sensitivity = NA_real_,
    specificity = NA_real_
  ))
}

thresholds <- seq(0, 1, by = 0.001)

mat <- sapply(thresholds, function(th) {
  pred_bin <- ifelse(pred >= th, 1, 0)

  TP <- sum(pred_bin == 1 & obs == 1)
  TN <- sum(pred_bin == 0 & obs == 0)
  FP <- sum(pred_bin == 1 & obs == 0)
  FN <- sum(pred_bin == 0 & obs == 1)

  sensitivity <- ifelse((TP + FN) == 0, NA_real_, TP / (TP + FN))
  specificity <- ifelse((TN + FP) == 0, NA_real_, TN / (TN + FP))
  TSS <- sensitivity + specificity - 1

  c(
    TSS = TSS,
    threshold = th,
```

```r
      sensitivity = sensitivity,
      specificity = specificity
    )
  })

  mat <- t(mat)
  best_i <- which.max(mat[, "TSS"])

  data.frame(
    TSS = mat[best_i, "TSS"],
    threshold = mat[best_i, "threshold"],
    sensitivity = mat[best_i, "sensitivity"],
    specificity = mat[best_i, "specificity"]
  )
}

calc_boyce <- function(obs, pred, n_bins = 10) {
  obs <- as.numeric(obs)
  pred <- as.numeric(pred)

  ok <- complete.cases(obs, pred)
  obs <- obs[ok]
  pred <- pred[ok]

  if (length(unique(obs)) < 2) return(NA_real_)
  if (sum(obs == 1) < 5) return(NA_real_)

  q <- unique(quantile(pred, probs = seq(0, 1, length.out = n_bins + 1), na.rm = TRUE))
```

```
  if (length(q) < 4) return(NA_real_)

  bin <- cut(pred, breaks = q, include.lowest = TRUE, labels = FALSE)

  all_freq <- as.numeric(table(factor(bin, levels = seq_len(length(q) - 1))))
  pres_freq <- as.numeric(table(factor(bin[obs == 1], levels = seq_len(length(q) - 1))))

  all_prop <- all_freq / sum(all_freq)
  pres_prop <- pres_freq / sum(pres_freq)

  ratio <- pres_prop / all_prop
  mids <- (q[-1] + q[-length(q)]) / 2

  ok2 <- is.finite(ratio) & is.finite(mids)

  if (sum(ok2) < 3) return(NA_real_)

  suppressWarnings(cor(mids[ok2], ratio[ok2], method = "spearman"))
}

evaluate_fold <- function(model_name, fold_id, obs, pred) {
  tss <- calc_tss(obs, pred)

  data.frame(
    Model = model_name,
    Fold = fold_id,
    AUC = calc_auc(obs, pred),
```

```r
    PR_AUC = calc_pr_auc(obs, pred),
    Boyce = calc_boyce(obs, pred),
    TSS = tss$TSS,
    Best_threshold = tss$threshold,
    Sensitivity = tss$sensitivity,
    Specificity = tss$specificity
  )
}

safe_try <- function(expr, label) {
  tryCatch(
    expr,
    error = function(e) {
      msg("FAILED: ", label, " | Error: ", e$message)
      NULL
    }
  )
}

make_formula <- function(response, predictors, smooth = FALSE) {
  if (!smooth) {
    as.formula(paste(response, "~", paste(predictors, collapse = " + ")))
  } else {
    terms <- ifelse(
      predictors == "land_cover",
      "land_cover",
      paste0("s(", predictors, ", k = 4)")
    )
```

```
    as.formula(paste(response, "~", paste(terms, collapse = " + ")))
  }
}

rescale01_raster <- function(r) {
  rng <- terra::global(r, range, na.rm = TRUE)
  mn <- rng[1, 1]
  mx <- rng[1, 2]

  if (!is.finite(mn) || !is.finite(mx) || mn == mx) return(r * NA)

  (r - mn) / (mx - mn)
}

rescale01_vec <- function(x) {
  rng <- range(x, na.rm = TRUE)

  if (!is.finite(rng[1]) || !is.finite(rng[2]) || rng[1] == rng[2]) {
    return(rep(NA_real_, length(x)))
  }

  (x - rng[1]) / (rng[2] - rng[1])
}

make_balanced_presence_spatial_folds <- function(data, k_target, min_presences) {
  presence_dat <- data %>% filter(pa == 1)
  background_dat <- data %>% filter(pa == 0)
```

```r
max_possible_k <- floor(nrow(presence_dat) / min_presences)

if (max_possible_k < 2) {
  fail("Too few presences for spatial validation. Reduce MIN_PRESENCES_PER_FOLD.")
}

k_use <- min(k_target, max_possible_k)

repeat {
  set.seed(123)

  km <- kmeans(
    scale(presence_dat[, c("longitude_dd", "latitude_dd")]),
    centers = k_use,
    nstart = 100
  )

  presence_dat$spatial_fold <- km$cluster

  pres_counts <- table(presence_dat$spatial_fold)

  if (min(pres_counts) >= min_presences || k_use == 2) break

  k_use <- k_use - 1
}

centroids <- presence_dat %>%
```

```
    group_by(spatial_fold) %>%
    summarise(
      lon_c = mean(longitude_dd),
      lat_c = mean(latitude_dd),
      .groups = "drop"
    )

  nearest_fold <- function(lon, lat, centroids) {
    d <- (centroids$lon_c - lon)^2 + (centroids$lat_c - lat)^2
    centroids$spatial_fold[which.min(d)]
  }

  background_dat$spatial_fold <- mapply(
    nearest_fold,
    background_dat$longitude_dd,
    background_dat$latitude_dd,
    MoreArgs = list(centroids = centroids)
  )

  out <- bind_rows(presence_dat, background_dat)
  out$spatial_fold <- as.integer(out$spatial_fold)

  attr(out, "k_used") <- k_use
  out
}

raster_to_df <- function(r) {
  df <- as.data.frame(r, xy = TRUE, na.rm = TRUE)
```

```r
  names(df)[3] <- "value"
  df
}

###############################################################
# 3. SELECT FOLDER AND CREATE OUTPUTS
###############################################################

main_dir <- get_folder()
setwd(main_dir)

safe_dir_create("outputs")
safe_dir_create("outputs/maps")
safe_dir_create("outputs/tables")
safe_dir_create("outputs/figures")
safe_dir_create("outputs/logs")
safe_dir_create("outputs/method_notes")

log_file <- file.path(
  "outputs/logs",
  paste0("swatwide_balanced_spatial_cv_log_", format(Sys.time(), "%Y%m%d_%H%M%S"), ".txt")
)

sink(log_file, split = TRUE)

on.exit({
  try(sink(), silent = TRUE)
}, add = TRUE)
```

```
msg("Working directory: ", getwd())
msg("Files found:")
print(list.files())

###############################################################
# 4. DETECT INPUT FILES
###############################################################

all_files <- list.files(getwd(), recursive = FALSE, full.names = TRUE)

occurrence_file <- find_one(
  all_files,
  "^black_bear_swatwide_presence_pseudoabsence_10126\\.csv$",
  "Swat-wide combined presence/pseudo-absence CSV"
)

bio_01_file <- find_one(all_files, "^bio[_-]?0?1\\.(tif|tiff|asc)$", "bio_01 raster")
bio_19_file <- find_one(all_files, "^bio[_-]?0?19\\.(tif|tiff|asc)$", "bio_19 raster")
land_cover_file <- find_one(all_files, "land[_-]?cover.*\\.(tif|tiff|asc)$", "land_cover raster")
settlements_file <- find_one(all_files, "settlement.*\\.(tif|tiff|asc)$", "settlements raster")
vrm_file <- find_one(all_files, "^vrm\\.(tif|tiff|asc)$", "VRM raster")
water_bodies_file <- find_one(all_files, "water.*\\.(tif|tiff|asc)$", "water_bodies raster")
shapefile_file <- find_one(all_files, "^swat\\.shp$", "Swat shapefile")

msg("Detected input files:")
print(data.frame(
  input = c(
```

```
    "occurrence", "bio_01", "bio_19", "land_cover",
    "settlements", "vrm", "water_bodies", "study_area"
  ),
  file = c(
    basename(occurrence_file), basename(bio_01_file), basename(bio_19_file),
    basename(land_cover_file), basename(settlements_file), basename(vrm_file),
    basename(water_bodies_file), basename(shapefile_file)
  )
))

################################################################
# 5. IMPORT OCCURRENCE / BACKGROUND DATA
################################################################

bb_data <- readr::read_csv(occurrence_file, show_col_types = FALSE)
bb_data <- as.data.frame(bb_data)
names(bb_data) <- make_valid_names(names(bb_data))

pa_col <- find_col(
  bb_data,
  c("pa", "presence_absence", "presenceabsence", "presence", "response"),
  "presence/pseudoabsence"
)

lon_col <- find_col(
  bb_data,
  c("longitude_dd", "longitude", "lon", "x", "long"),
  "longitude"
```

```
)

lat_col <- find_col(
  bb_data,
  c("latitude_dd", "latitude", "lat", "y"),
  "latitude"
)

bb_data$pa <- as.numeric(bb_data[[pa_col]])
bb_data$longitude_dd <- as.numeric(bb_data[[lon_col]])
bb_data$latitude_dd <- as.numeric(bb_data[[lat_col]])

bb_data <- bb_data %>%
  filter(!is.na(pa), !is.na(longitude_dd), !is.na(latitude_dd)) %>%
  filter(pa %in% c(0, 1))

if (length(unique(bb_data$pa)) < 2) fail("Data must contain both presence and pseudo-
absence/background.")

msg("Original class counts:")
print(table(bb_data$pa))

################################################################
# 6. IMPORT STUDY AREA AND OPTIONAL ACCESSIBLE BACKGROUND
################################################################

study_area <- terra::vect(shapefile_file)
```

```
if (USE_ACCESSIBLE_AREA_BACKGROUND) {
 msg("Applying accessible-area background filter.")

 bb_sf <- sf::st_as_sf(
  bb_data,
  coords = c("longitude_dd", "latitude_dd"),
  crs = 4326,
  remove = FALSE
 )

 bb_utm <- sf::st_transform(bb_sf, 32643)
 study_sf <- sf::st_as_sf(study_area)
 study_utm <- sf::st_transform(study_sf, 32643)

 pres_buf <- bb_utm %>%
  filter(pa == 1) %>%
  sf::st_buffer(dist = ACCESSIBLE_BUFFER_KM * 1000) %>%
  sf::st_union()

 accessible_area <- sf::st_intersection(pres_buf, sf::st_union(study_utm))

 bg <- bb_utm %>% filter(pa == 0)
 pres <- bb_utm %>% filter(pa == 1)

 bg_inside <- sf::st_intersects(bg, accessible_area, sparse = FALSE)[, 1]
 bg_keep <- bg[bg_inside, ]

 if (nrow(bg_keep) > TARGET_BACKGROUND_N) {
```

```
    set.seed(999)
    bg_keep <- bg_keep[sample(seq_len(nrow(bg_keep)), TARGET_BACKGROUND_N), ]
  }

  bb_data <- bind_rows(
    sf::st_drop_geometry(pres),
    sf::st_drop_geometry(bg_keep)
  )

  msg("Class counts after accessible-area background filtering:")
  print(table(bb_data$pa))
}

pseudoabsence_summary <- data.frame(
  total_records = nrow(bb_data),
  presence_records = sum(bb_data$pa == 1),
  pseudoabsence_background_records = sum(bb_data$pa == 0),
  pseudoabsence_to_presence_ratio = round(sum(bb_data$pa == 0) / sum(bb_data$pa == 1), 2),
  background_domain = ifelse(
    USE_ACCESSIBLE_AREA_BACKGROUND,
    paste0("Accessible area buffer around presences: ", ACCESSIBLE_BUFFER_KM, " km"),
    "Full Swat-wide modelling domain"
  ),
  interpretation = "Pseudo-absence/background points represent available environmental conditions, not confirmed true absences."
)

write_csv(
```

```
  pseudoabsence_summary,
  "outputs/tables/swatwide_pseudoabsence_background_summary_balanced_cv.csv"
)

###############################################################
# 7. IMPORT AND ALIGN RASTERS
###############################################################

bio_01 <- terra::rast(bio_01_file)
bio_19 <- terra::rast(bio_19_file)
land_cover <- terra::rast(land_cover_file)
settlements <- terra::rast(settlements_file)
vrm <- terra::rast(vrm_file)
water_bodies <- terra::rast(water_bodies_file)

names(bio_01) <- "bio_01"
names(bio_19) <- "bio_19"
names(land_cover) <- "land_cover"
names(settlements) <- "settlements"
names(vrm) <- "vrm"
names(water_bodies) <- "water_bodies"

template <- bio_01

bio_19 <- terra::resample(bio_19, template, method = "bilinear")
land_cover <- terra::resample(land_cover, template, method = "near")
settlements <- terra::resample(settlements, template, method = "bilinear")
vrm <- terra::resample(vrm, template, method = "bilinear")
```

```
water_bodies <- terra::resample(water_bodies, template, method = "bilinear")

env_stack <- c(
  bio_01,
  bio_19,
  land_cover,
  settlements,
  vrm,
  water_bodies
)

names(env_stack) <- c(
  "bio_01", "bio_19", "land_cover",
  "settlements", "vrm", "water_bodies"
)

if (is.na(terra::crs(env_stack))) fail("Raster CRS is missing.")

study_area_projected <- terra::project(study_area, terra::crs(env_stack))

env_crop <- terra::crop(env_stack, study_area_projected)
env_mask <- terra::mask(env_crop, study_area_projected)

if (terra::ncell(env_mask) == 0) fail("Masked raster has zero cells.")

terra::writeRaster(
  env_mask,
  "outputs/maps/environmental_stack_masked_swatwide_balanced_cv.tif",
```

```
  overwrite = TRUE
)

##################################################################
# 8. EXTRACT RASTER VALUES
##################################################################

bb_points <- terra::vect(
  bb_data,
  geom = c("longitude_dd", "latitude_dd"),
  crs = "EPSG:4326"
)

bb_points_projected <- terra::project(bb_points, terra::crs(env_mask))

env_values <- terra::extract(env_mask, bb_points_projected)

model_data <- cbind(bb_data, env_values[, -1, drop = FALSE])
model_data[model_data == -9999] <- NA
model_data[model_data == -3.4e+38] <- NA

all_predictors <- c(
  "bio_01", "bio_19", "land_cover",
  "settlements", "vrm", "water_bodies"
)

model_data_clean <- model_data %>%
  dplyr::select(any_of(c("pa", "longitude_dd", "latitude_dd", all_predictors))) %>%
```

```
  tidyr::drop_na(all_of(c("pa", all_predictors)))

model_data_clean$pa <- as.numeric(model_data_clean$pa)
model_data_clean$land_cover <- as.numeric(model_data_clean$land_cover)

model_data_clean$pa_factor <- factor(
  ifelse(model_data_clean$pa == 1, "presence", "absence"),
  levels = c("absence", "presence")
)

if (length(unique(model_data_clean$pa)) < 2) {
  fail("After raster extraction, only one class remains.")
}

write_csv(
  model_data_clean,
  "outputs/tables/swatwide_final_model_dataset_before_collinearity_balanced_cv.csv"
)

############################################################
# 9. COLLINEARITY CHECK
############################################################

continuous_predictors <- setdiff(all_predictors, "land_cover")

cor_mat <- cor(
  model_data_clean[, continuous_predictors, drop = FALSE],
  use = "pairwise.complete.obs"
```

```
)

write.csv(
  cor_mat,
  "outputs/tables/swatwide_predictor_correlation_matrix_balanced_cv.csv",
  row.names = TRUE
)

remove_predictors <- character(0)

if (length(continuous_predictors) > 1) {
  cm_abs <- abs(cor_mat)
  diag(cm_abs) <- 0

  while (max(cm_abs, na.rm = TRUE) >= COLLINEARITY_CUTOFF) {
    high_pair <- which(cm_abs == max(cm_abs, na.rm = TRUE), arr.ind = TRUE)[1, ]

    p1 <- rownames(cm_abs)[high_pair[1]]
    p2 <- colnames(cm_abs)[high_pair[2]]

    mean_cor_p1 <- mean(cm_abs[p1, ], na.rm = TRUE)
    mean_cor_p2 <- mean(cm_abs[p2, ], na.rm = TRUE)

    drop_p <- ifelse(mean_cor_p1 >= mean_cor_p2, p1, p2)

    remove_predictors <- unique(c(remove_predictors, drop_p))

    cm_abs <- cm_abs[
```

```
      setdiff(rownames(cm_abs), drop_p),
      setdiff(colnames(cm_abs), drop_p),
      drop = FALSE
    ]

    if (nrow(cm_abs) <= 1) break
  }
}

predictor_names <- setdiff(all_predictors, remove_predictors)

if (length(predictor_names) < 2) fail("Too few predictors remain.")

collinearity_decision <- data.frame(
  predictor = all_predictors,
  retained = all_predictors %in% predictor_names,
  reason = ifelse(
    all_predictors %in% remove_predictors,
    paste0("Removed because |r| >= ", COLLINEARITY_CUTOFF),
    paste0("Retained because |r| < ", COLLINEARITY_CUTOFF, " or categorical")
  )
)

write_csv(
  collinearity_decision,
  "outputs/tables/swatwide_collinearity_decision_balanced_cv.csv"
)
```

```
model_data_clean <- model_data_clean %>%
  dplyr::select(any_of(c(
    "pa", "pa_factor", "longitude_dd", "latitude_dd", predictor_names
  )))

write_csv(
  model_data_clean,
  "outputs/tables/swatwide_final_model_dataset_used_balanced_cv.csv"
)

msg("Final predictors:")
print(predictor_names)

############################################################
# 10. BALANCED SPATIAL K-MEANS CROSS-VALIDATION
############################################################

model_data_clean <- make_balanced_presence_spatial_folds(
  model_data_clean,
  k_target = K_FOLDS_TARGET,
  min_presences = MIN_PRESENCES_PER_FOLD
)

K_FOLDS_USED <- attr(model_data_clean, "k_used")

fold_table <- table(model_data_clean$spatial_fold, model_data_clean$pa)

write.csv(
```

```
  as.data.frame.matrix(fold_table),
  "outputs/tables/swatwide_balanced_spatial_cv_fold_class_counts.csv"
)

msg("Balanced spatial fold class counts:")
print(fold_table)

writeLines(
  c(
    "Balanced spatial cross-validation note:",
    "",
    paste0("Target folds: ", K_FOLDS_TARGET),
    paste0("Folds used: ", K_FOLDS_USED),
    paste0("Minimum required presences per fold: ", MIN_PRESENCES_PER_FOLD),
    "",
    "Presence records were partitioned into spatial clusters using k-means on geographic coordinates.",
    "The number of folds was reduced automatically if any fold contained too few presences.",
    "Pseudo-absence/background records were assigned to the nearest presence-fold centroid.",
    "This prevents invalid folds with very low presence counts and provides more defensible spatial validation than the previous 10-block run."
  ),
  "outputs/method_notes/balanced_spatial_cv_justification.txt"
)

############################################################
# 11. MODEL FUNCTIONS
############################################################
```

```
fit_predict_one_fold <- function(model_name, train_data, test_data, predictors) {
  f_numeric <- make_formula("pa", predictors, smooth = FALSE)
  f_factor <- make_formula("pa_factor", predictors, smooth = FALSE)
  f_gam <- make_formula("pa", predictors, smooth = TRUE)

  if (model_name == "GLM") {
    fit <- glm(f_numeric, data = train_data, family = binomial)
    pred <- predict(fit, newdata = test_data, type = "response")
    return(list(fit = fit, pred = pred))
  }

  if (model_name == "GAM") {
    fit <- mgcv::gam(f_gam, data = train_data, family = binomial, method = "REML")
    pred <- predict(fit, newdata = test_data, type = "response")
    return(list(fit = fit, pred = pred))
  }

  if (model_name == "Random_Forest") {
    fit <- randomForest(
      f_factor,
      data = train_data,
      ntree = RF_TREES,
      importance = TRUE
    )

    pred <- predict(fit, newdata = test_data, type = "prob")[, "presence"]
    return(list(fit = fit, pred = pred))
  }
```

```r
if (model_name == "GBM") {
  fit <- gbm(
    formula = f_numeric,
    data = train_data,
    distribution = "bernoulli",
    n.trees = GBM_TREES,
    interaction.depth = 3,
    shrinkage = 0.01,
    bag.fraction = 0.7,
    train.fraction = 1,
    verbose = FALSE
  )

  pred <- predict(
    fit,
    newdata = test_data,
    n.trees = GBM_TREES,
    type = "response"
  )

  return(list(fit = fit, pred = pred))
}

if (model_name == "SVM") {
  fit <- svm(
    f_factor,
    data = train_data,
```

```
    type = "C-classification",
    kernel = "radial",
    probability = TRUE,
    scale = TRUE
  )

  cls <- predict(fit, newdata = test_data, probability = TRUE)
  pr <- attr(cls, "probabilities")
  pred <- pr[, "presence"]

  return(list(fit = fit, pred = pred))
}

if (model_name == "Maxnet") {
  set.seed(456)

  inner_idx <- sample(
    seq_len(nrow(train_data)),
    size = floor(0.80 * nrow(train_data))
  )

  inner_train <- train_data[inner_idx, ]
  inner_valid <- train_data[-inner_idx, ]

  classes_grid <- c("l", "lq", "lqh", "lqph")
  reg_grid <- c(0.5, 1, 1.5, 2, 3, 4)

  best_auc <- -Inf
```

```
best_classes <- "lqph"
best_reg <- 1

for (cl in classes_grid) {
  for (rg in reg_grid) {
    tmp <- tryCatch({
      x_in <- inner_train[, predictors, drop = FALSE]
      p_in <- inner_train$pa

      m <- maxnet(
        p = p_in,
        data = x_in,
        f = maxnet.formula(p_in, x_in, classes = cl),
        regmult = rg
      )

      pv <- predict(
        m,
        newdata = inner_valid[, predictors, drop = FALSE],
        type = "cloglog"
      )

      auc <- calc_auc(inner_valid$pa, pv)

      list(auc = auc, classes = cl, reg = rg)
    }, error = function(e) NULL)

    if (!is.null(tmp) && is.finite(tmp$auc) && tmp$auc > best_auc) {
```

```
      best_auc <- tmp$auc
      best_classes <- tmp$classes
      best_reg <- tmp$reg
    }
  }
}

fit <- maxnet(
  p = train_data$pa,
  data = train_data[, predictors, drop = FALSE],
  f = maxnet.formula(
    train_data$pa,
    train_data[, predictors, drop = FALSE],
    classes = best_classes
  ),
  regmult = best_reg
)

pred <- predict(
  fit,
  newdata = test_data[, predictors, drop = FALSE],
  type = "cloglog"
)

attr(fit, "best_classes") <- best_classes
attr(fit, "best_regmult") <- best_reg

return(list(fit = fit, pred = pred))
```

```
  }

  fail("Unknown model: ", model_name)
}

#################################################################
# 12. CROSS-VALIDATION
#################################################################

models_to_run <- c("GLM", "GAM", "Random_Forest", "GBM", "Maxnet")

if (RUN_SVM) models_to_run <- c(models_to_run, "SVM")

fold_ids <- sort(unique(model_data_clean$spatial_fold))

eval_rows <- list()
pred_store <- list()

for (m in models_to_run) {
  msg("Cross-validating model: ", m)

  for (fold in fold_ids) {
    train_data <- model_data_clean %>% filter(spatial_fold != fold)
    test_data <- model_data_clean %>% filter(spatial_fold == fold)

    if (length(unique(train_data$pa)) < 2 || length(unique(test_data$pa)) < 2) {
      msg("Skipping ", m, " fold ", fold, " because train/test has one class.")
      next
```

```
    }

    res <- safe_try(
      fit_predict_one_fold(m, train_data, test_data, predictor_names),
      paste0(m, " fold ", fold)
    )

    if (!is.null(res)) {
      ev <- evaluate_fold(m, fold, test_data$pa, res$pred)
      eval_rows[[length(eval_rows) + 1]] <- ev

      pred_store[[length(pred_store) + 1]] <- data.frame(
        Model = m,
        Fold = fold,
        observed = test_data$pa,
        predicted = as.numeric(res$pred)
      )
    }
  }
}

eval_by_fold <- bind_rows(eval_rows)

write_csv(
  eval_by_fold,
  "outputs/tables/swatwide_model_evaluation_by_balanced_spatial_fold.csv"
)
```

```
eval_table <- eval_by_fold %>%
  group_by(Model) %>%
  summarise(
    n_folds = n(),
    Mean_AUC = mean(AUC, na.rm = TRUE),
    SD_AUC = sd(AUC, na.rm = TRUE),
    Mean_PR_AUC = mean(PR_AUC, na.rm = TRUE),
    SD_PR_AUC = sd(PR_AUC, na.rm = TRUE),
    Mean_Boyce = mean(Boyce, na.rm = TRUE),
    SD_Boyce = sd(Boyce, na.rm = TRUE),
    Mean_TSS = mean(TSS, na.rm = TRUE),
    SD_TSS = sd(TSS, na.rm = TRUE),
    Mean_threshold = mean(Best_threshold, na.rm = TRUE),
    Mean_sensitivity = mean(Sensitivity, na.rm = TRUE),
    Mean_specificity = mean(Specificity, na.rm = TRUE),
    CV_instability_flag = ifelse(
      SD_AUC > 0.15 | SD_TSS > 0.25,
      "High spatial variability",
      "Acceptable"
    ),
    .groups = "drop"
  ) %>%
  arrange(desc(Mean_AUC))

write_csv(
  eval_table,
  "outputs/tables/swatwide_model_evaluation_balanced_spatial_cv_summary.csv"
)
```

```
cv_predictions <- bind_rows(pred_store)

write_csv(
  cv_predictions,
  "outputs/tables/swatwide_cross_validated_predictions_balanced_spatial_cv.csv"
)

msg("Balanced spatial CV summary:")
print(eval_table)

#################################################################
# 13. PERFORMANCE PLOTS
#################################################################

perf_long <- eval_table %>%
  dplyr::select(Model, Mean_AUC, SD_AUC, Mean_TSS, SD_TSS) %>%
  tidyr::pivot_longer(
    cols = c(Mean_AUC, Mean_TSS),
    names_to = "Metric",
    values_to = "Mean"
  ) %>%
  mutate(
    SD = ifelse(Metric == "Mean_AUC", SD_AUC, SD_TSS),
    Metric = ifelse(Metric == "Mean_AUC", "AUC", "TSS")
  )

p_perf <- ggplot(perf_long, aes(x = reorder(Model, Mean), y = Mean, fill = Metric)) +
```

```
  geom_col(position = position_dodge(width = 0.75), width = 0.65, colour = "grey25") +
  geom_errorbar(
    aes(ymin = pmax(0, Mean - SD), ymax = pmin(1, Mean + SD)),
    position = position_dodge(width = 0.75),
    width = 0.20,
    linewidth = 0.6
  ) +
  scale_fill_viridis_d(option = "D", end = 0.85, name = "Metric") +
  coord_flip() +
  scale_y_continuous(limits = c(0, 1), breaks = seq(0, 1, 0.2)) +
  theme_bw(base_size = 14) +
  labs(
    title = "Balanced spatial cross-validation performance",
    x = "Model",
    y = "Mean score ± SD"
  ) +
  theme(
    plot.title = element_text(face = "bold", hjust = 0.5),
    legend.position = "top"
  )

ggsave(
  "outputs/figures/model_performance_AUC_TSS_balanced_spatial_cv.png",
  p_perf,
  width = 9,
  height = 6,
  dpi = 300
)
```

```
##############################################################
# 14. ENSEMBLE MODEL SELECTION
##############################################################

selected_models <- eval_table %>%
  filter(Mean_AUC >= ENSEMBLE_MIN_AUC, Mean_TSS >= ENSEMBLE_MIN_TSS) %>%
  pull(Model)

if (length(selected_models) < 2) {
  msg("Fewer than two models passed AUC/TSS rule. Using top models by Mean_AUC.")

  selected_models <- eval_table %>%
    arrange(desc(Mean_AUC)) %>%
    slice_head(n = min(ENSEMBLE_TOP_N_FALLBACK, n())) %>%
    pull(Model)
}

ensemble_decision <- eval_table %>%
  mutate(
    Included_in_ensemble = Model %in% selected_models,
    Rule = paste0(
      "Preferred rule: Mean_AUC >= ", ENSEMBLE_MIN_AUC,
      " and Mean_TSS >= ", ENSEMBLE_MIN_TSS,
      "; fallback: top ", ENSEMBLE_TOP_N_FALLBACK, " by Mean_AUC"
    )
  )
```

```r
write_csv(
  ensemble_decision,
  "outputs/tables/swatwide_ensemble_model_selection_decision_balanced_cv.csv"
)

msg("Selected ensemble models:")
print(selected_models)

#################################################################
# 15. FIT FINAL MODELS ON FULL DATASET
#################################################################

fit_final_model <- function(model_name, data, predictors) {
  f_numeric <- make_formula("pa", predictors, smooth = FALSE)
  f_factor <- make_formula("pa_factor", predictors, smooth = FALSE)
  f_gam <- make_formula("pa", predictors, smooth = TRUE)

  if (model_name == "GLM") {
    return(glm(f_numeric, data = data, family = binomial))
  }

  if (model_name == "GAM") {
    return(mgcv::gam(f_gam, data = data, family = binomial, method = "REML"))
  }

  if (model_name == "Random_Forest") {
    return(randomForest(
      f_factor,
```

```
    data = data,
    ntree = RF_TREES,
    importance = TRUE
  ))
}

if (model_name == "GBM") {
  return(gbm(
    formula = f_numeric,
    data = data,
    distribution = "bernoulli",
    n.trees = GBM_TREES,
    interaction.depth = 3,
    shrinkage = 0.01,
    bag.fraction = 0.7,
    train.fraction = 1,
    verbose = FALSE
  ))
}

if (model_name == "SVM") {
  return(svm(
    f_factor,
    data = data,
    type = "C-classification",
    kernel = "radial",
    probability = TRUE,
    scale = TRUE
```

```r
  ))
}

if (model_name == "Maxnet") {
  set.seed(789)

  inner_idx <- sample(
    seq_len(nrow(data)),
    size = floor(0.80 * nrow(data))
  )

  inner_train <- data[inner_idx, ]
  inner_valid <- data[-inner_idx, ]

  classes_grid <- c("l", "lq", "lqh", "lqph")
  reg_grid <- c(0.5, 1, 1.5, 2, 3, 4)

  best_auc <- -Inf
  best_classes <- "lqph"
  best_reg <- 1

  for (cl in classes_grid) {
    for (rg in reg_grid) {
      tmp <- tryCatch({
        x_in <- inner_train[, predictors, drop = FALSE]
        p_in <- inner_train$pa

        m <- maxnet(
```

```
      p = p_in,
      data = x_in,
      f = maxnet.formula(p_in, x_in, classes = cl),
      regmult = rg
    )

    pv <- predict(
      m,
      newdata = inner_valid[, predictors, drop = FALSE],
      type = "cloglog"
    )

    auc <- calc_auc(inner_valid$pa, pv)

    list(auc = auc, classes = cl, reg = rg)
  }, error = function(e) NULL)

  if (!is.null(tmp) && is.finite(tmp$auc) && tmp$auc > best_auc) {
    best_auc <- tmp$auc
    best_classes <- tmp$classes
    best_reg <- tmp$reg
  }
 }
}

m <- maxnet(
 p = data$pa,
 data = data[, predictors, drop = FALSE],
```

```
      f = maxnet.formula(
        data$pa,
        data[, predictors, drop = FALSE],
        classes = best_classes
      ),
      regmult = best_reg
    )

    attr(m, "best_classes") <- best_classes
    attr(m, "best_regmult") <- best_reg

    return(m)
  }

  fail("Unknown model.")
}

final_models <- list()

for (m in unique(eval_table$Model)) {
  msg("Fitting final model: ", m)

  final_models[[m]] <- safe_try(
    fit_final_model(m, model_data_clean, predictor_names),
    paste0("final ", m)
  )
}
```

```
############################################################
# 16. VARIABLE IMPORTANCE PLOTS
############################################################

if (!is.null(final_models[["Random_Forest"]])) {
  rf_imp <- data.frame(
    Variable = rownames(randomForest::importance(final_models[["Random_Forest"]])),
    Importance = randomForest::importance(final_models[["Random_Forest"]])[, "MeanDecreaseGini"]
  ) %>%
    arrange(Importance) %>%
    mutate(Variable = factor(Variable, levels = Variable))

  write_csv(
    rf_imp,
    "outputs/tables/swatwide_random_forest_variable_importance_balanced_cv.csv"
  )

  p_rf <- ggplot(rf_imp, aes(x = Variable, y = Importance, fill = Importance)) +
    geom_col(width = 0.72, colour = "grey20", linewidth = 0.25) +
    coord_flip() +
    scale_fill_viridis_c(
      option = "C",
      direction = -1,
      name = "Importance"
    ) +
    theme_minimal(base_size = 15) +
    labs(
      title = "Random Forest variable importance",
```

```
    subtitle = "Mean decrease in Gini index",
    x = "Environmental predictor",
    y = "Importance"
   ) +
   theme(
    plot.title = element_text(face = "bold", size = 18),
    plot.subtitle = element_text(size = 12),
    axis.title = element_text(face = "bold"),
    panel.grid.major.y = element_blank(),
    panel.grid.minor = element_blank(),
    legend.position = "right"
   )

  ggsave(
   "outputs/figures/random_forest_variable_importance_coloured_balanced_cv.png",
   p_rf,
   width = 9,
   height = 6,
   dpi = 300
  )
}

if (!is.null(final_models[["GBM"]])) {
  gbm_imp <- summary(final_models[["GBM"]], n.trees = GBM_TREES, plotit = FALSE)

  write_csv(
   gbm_imp,
   "outputs/tables/swatwide_gbm_variable_importance_balanced_cv.csv"
```

```
 )
}

#################################################################
# 17. PREDICT MAPS
#################################################################

env_pred <- env_mask[[predictor_names]]
names(env_pred) <- predictor_names

predict_model_map <- function(model_name, model, raster_stack) {
  if (is.null(model)) return(NULL)

  msg("Predicting map for ", model_name)

  if (model_name == "GLM") {
    return(terra::predict(
      raster_stack,
      model,
      fun = function(m, d) predict(m, newdata = as.data.frame(d), type = "response"),
      na.rm = TRUE
    ))
  }

  if (model_name == "GAM") {
    return(terra::predict(
      raster_stack,
      model,
```

```
    fun = function(m, d) predict(m, newdata = as.data.frame(d), type = "response"),
    na.rm = TRUE
  ))
}

if (model_name == "Random_Forest") {
  return(terra::predict(
    raster_stack,
    model,
    fun = function(m, d) predict(m, newdata = as.data.frame(d), type = "prob")[, "presence"],
    na.rm = TRUE
  ))
}

if (model_name == "GBM") {
  return(terra::predict(
    raster_stack,
    model,
    fun = function(m, d) predict(m, newdata = as.data.frame(d), n.trees = GBM_TREES, type =
"response"),
    na.rm = TRUE
  ))
}

if (model_name == "SVM") {
  return(terra::predict(
    raster_stack,
    model,
```

```
      fun = function(m, d) {
        out <- predict(m, newdata = as.data.frame(d), probability = TRUE)
        attr(out, "probabilities")[, "presence"]
      },
      na.rm = TRUE
    ))
  }

  if (model_name == "Maxnet") {
    return(terra::predict(
      raster_stack,
      model,
      fun = function(m, d) predict(m, newdata = as.data.frame(d), type = "cloglog"),
      na.rm = TRUE
    ))
  }

  NULL
}

map_list_raw <- list()
map_list_rescaled <- list()

for (m in names(final_models)) {
  mp <- safe_try(
    predict_model_map(m, final_models[[m]], env_pred),
    paste0("map prediction ", m)
  )
```

```
  if (!is.null(mp)) {
    names(mp) <- m

    mp_rescaled <- rescale01_raster(mp)
    names(mp_rescaled) <- m

    map_list_raw[[m]] <- mp
    map_list_rescaled[[m]] <- mp_rescaled

    terra::writeRaster(
      mp,
      paste0("outputs/maps/swatwide_suitability_raw_", m, "_balanced_cv.tif"),
      overwrite = TRUE
    )

    terra::writeRaster(
      mp_rescaled,
      paste0("outputs/maps/swatwide_suitability_rescaled_", m, "_balanced_cv.tif"),
      overwrite = TRUE
    )
  }
}

if (length(map_list_rescaled) == 0) fail("No maps produced.")

############################################################
# 18. ENSEMBLE MAP — FIXED FALLBACK LOGIC
```

```
############################################################

# Identify models for which maps were successfully produced
available_model_maps <- intersect(eval_table$Model, names(map_list_rescaled))

if (length(available_model_maps) < 2) {
  fail("Fewer than two model prediction maps are available. Cannot build ensemble.")
}

# Preferred model selection rule
preferred_selected <- eval_table %>%
  dplyr::filter(
    Model %in% available_model_maps,
    Mean_AUC >= ENSEMBLE_MIN_AUC,
    Mean_TSS >= ENSEMBLE_MIN_TSS
  ) %>%
  dplyr::arrange(dplyr::desc(Mean_AUC)) %>%
  dplyr::pull(Model)

# Correct fallback rule
if (length(preferred_selected) >= 2) {
  available_selected <- preferred_selected
  ensemble_rule_used <- paste0(
    "Models selected using AUC >= ",
    ENSEMBLE_MIN_AUC,
    " and TSS >= ",
    ENSEMBLE_MIN_TSS
  )
```

```
} else {
  msg("Fewer than two models passed the preferred AUC/TSS rule. Using top available models by Mean_AUC.")

  ranked_available <- eval_table %>%
    dplyr::filter(Model %in% available_model_maps) %>%
    dplyr::arrange(dplyr::desc(Mean_AUC))

  n_take <- min(ENSEMBLE_TOP_N_FALLBACK, nrow(ranked_available))

  available_selected <- ranked_available$Model[seq_len(n_take)]

  ensemble_rule_used <- paste0(
    "Fallback used: top ",
    n_take,
    " available models by Mean_AUC"
  )
}

if (length(available_selected) < 2) {
  fail("Ensemble requires at least two selected models. Available selected models: ",
       paste(available_selected, collapse = ", "))
}

msg("Final selected models for ensemble:")
print(available_selected)

# Build SpatRaster stack safely from list
```

```r
selected_maps <- map_list_rescaled[[available_selected[1]]]


if (length(available_selected) > 1) {
  for (i in 2:length(available_selected)) {
    selected_maps <- c(selected_maps, map_list_rescaled[[available_selected[i]]])
  }
}


names(selected_maps) <- available_selected


# AUC weights
selected_weights <- eval_table$Mean_AUC[match(available_selected, eval_table$Model)]
selected_weights[is.na(selected_weights)] <- mean(eval_table$Mean_AUC, na.rm = TRUE)


if (sum(selected_weights, na.rm = TRUE) <= 0) {
  selected_weights <- rep(1 / length(available_selected), length(available_selected))
} else {
  selected_weights <- selected_weights / sum(selected_weights, na.rm = TRUE)
}


weight_table <- data.frame(
  Model = available_selected,
  AUC_weight = selected_weights,
  Selection_rule = ensemble_rule_used
)


write_csv(
  weight_table,
```

```
  "outputs/tables/ensemble_auc_weights.csv"
)

ensemble_decision_fixed <- eval_table %>%
  dplyr::mutate(
    Included_in_ensemble = Model %in% available_selected,
    Selection_rule = ensemble_rule_used
  )

write_csv(
  ensemble_decision_fixed,
  "outputs/tables/ensemble_model_selection_decision.csv"
)

# Weighted ensemble
ensemble_map <- terra::app(selected_maps, fun = function(x) {
  if (all(is.na(x))) return(NA_real_)
  sum(x * selected_weights, na.rm = TRUE)
})

ensemble_map <- rescale01_raster(ensemble_map)
names(ensemble_map) <- "Relative_suitability"

# Model uncertainty
uncertainty_map <- terra::app(selected_maps, fun = sd, na.rm = TRUE)
names(uncertainty_map) <- "Prediction_uncertainty"

terra::writeRaster(
```

```r
  ensemble_map,
  "outputs/maps/ensemble_relative_suitability.tif",
  overwrite = TRUE
)

terra::writeRaster(
  uncertainty_map,
  "outputs/maps/prediction_uncertainty.tif",
  overwrite = TRUE
)

############################################################
# 19. CLASSIFICATION AND AREA
############################################################

ens_vals <- terra::values(ensemble_map, mat = FALSE)
ens_vals <- ens_vals[is.finite(ens_vals)]

if (length(ens_vals) == 0) {
  fail("No finite ensemble suitability values found.")
}

q_low_mod <- as.numeric(quantile(ens_vals, probs = LOW_MOD_Q, na.rm = TRUE))
q_mod_high <- as.numeric(quantile(ens_vals, probs = MOD_HIGH_Q, na.rm = TRUE))

quantile_thresholds <- data.frame(
  Threshold = c("Low to moderate", "Moderate to high"),
  Quantile = c(LOW_MOD_Q, MOD_HIGH_Q),
```

```r
  Suitability_value = c(q_low_mod, q_mod_high)
)

write_csv(
  quantile_thresholds,
  "outputs/tables/quantile_suitability_thresholds.csv"
)

rcl_quant <- matrix(c(
  -Inf, q_low_mod, 1,
  q_low_mod, q_mod_high, 2,
  q_mod_high, Inf, 3
), ncol = 3, byrow = TRUE)

ensemble_class_quantile <- terra::classify(
  ensemble_map,
  rcl = rcl_quant,
  include.lowest = TRUE
)

names(ensemble_class_quantile) <- "Suitability_class"

terra::writeRaster(
  ensemble_class_quantile,
  "outputs/maps/suitability_classes_quantile.tif",
  overwrite = TRUE
)
```

```
# TSS threshold
selected_thresholds <- eval_table$Mean_threshold[match(available_selected, eval_table$Model)]
selected_thresholds[is.na(selected_thresholds)] <- mean(eval_table$Mean_threshold, na.rm = TRUE)

ensemble_tss_threshold <- sum(selected_thresholds * selected_weights, na.rm = TRUE)

tss_binary_map <- ensemble_map >= ensemble_tss_threshold
names(tss_binary_map) <- "Suitable_habitat"

terra::writeRaster(
  tss_binary_map,
  "outputs/maps/suitable_habitat_TSS_threshold.tif",
  overwrite = TRUE
)

write_csv(
  data.frame(TSS_threshold = ensemble_tss_threshold),
  "outputs/tables/TSS_binary_threshold.csv"
)

# Area calculations
cell_area_km2 <- terra::cellSize(ensemble_map, unit = "km")
names(cell_area_km2) <- "Area_km2"

area_class <- terra::zonal(
  cell_area_km2,
  ensemble_class_quantile,
  fun = "sum",
```

```r
  na.rm = TRUE
)

names(area_class) <- c("Suitability_class", "Area_km2")

area_class$Class_name <- dplyr::case_when(
  area_class$Suitability_class == 1 ~ "Low suitability",
  area_class$Suitability_class == 2 ~ "Moderate suitability",
  area_class$Suitability_class == 3 ~ "High suitability",
  TRUE ~ "Unknown"
)

area_class <- area_class %>%
  dplyr::select(Suitability_class, Class_name, Area_km2)

area_class$Percent_area <- 100 * area_class$Area_km2 / sum(area_class$Area_km2, na.rm = TRUE)

write_csv(
  area_class,
  "outputs/tables/suitability_class_area_km2.csv"
)

area_binary <- terra::zonal(
  cell_area_km2,
  tss_binary_map,
  fun = "sum",
  na.rm = TRUE
)
```

```
names(area_binary) <- c("Suitable_binary", "Area_km2")

area_binary$Class_name <- ifelse(
  area_binary$Suitable_binary == 1,
  "Suitable",
  "Unsuitable"
)

area_binary$Percent_area <- 100 * area_binary$Area_km2 / sum(area_binary$Area_km2, na.rm =
TRUE)

write_csv(
  area_binary,
  "outputs/tables/suitable_unsuitable_area_km2.csv"
)

############################################################
# 20. PUBLICATION-QUALITY MAPS
############################################################

study_sf_plot <- sf::st_as_sf(study_area_projected)

points_df <- model_data_clean %>%
  dplyr::filter(pa == 1) %>%
  dplyr::select(longitude_dd, latitude_dd)

ensemble_df <- raster_to_df(ensemble_map)
```

```r
uncertainty_df <- raster_to_df(uncertainty_map)
class_df <- raster_to_df(ensemble_class_quantile)
binary_df <- raster_to_df(tss_binary_map)

class_df$value <- factor(
  class_df$value,
  levels = c(1, 2, 3),
  labels = c("Low", "Moderate", "High")
)

binary_df$value <- ifelse(as.logical(binary_df$value), "Suitable", "Unsuitable")

binary_df$value <- factor(
  binary_df$value,
  levels = c("Unsuitable", "Suitable")
)

# 20.1 Continuous suitability map
p_ensemble <- ggplot() +
  geom_raster(data = ensemble_df, aes(x = x, y = y, fill = value)) +
  geom_sf(data = study_sf_plot, fill = NA, colour = "black", linewidth = 0.45) +
  geom_point(
    data = points_df,
    aes(x = longitude_dd, y = latitude_dd),
    colour = "red",
    size = 1.1,
    alpha = 0.85
  ) +
```

```
  scale_fill_viridis_c(
    option = "C",
    name = "Relative\nsuitability",
    limits = c(0, 1),
    breaks = seq(0, 1, 0.2)
  ) +
  coord_sf(expand = FALSE) +
  theme_minimal(base_size = 14) +
  labs(
    title = "Ensemble habitat suitability",
    x = "Longitude",
    y = "Latitude"
  ) +
  theme(
    plot.title = element_text(face = "bold", size = 18, hjust = 0.5),
    legend.title = element_text(face = "bold"),
    legend.position = "right",
    panel.grid.major = element_line(colour = "grey85", linewidth = 0.2)
  )

ggsave(
  "outputs/figures/ensemble_habitat_suitability.png",
  p_ensemble,
  width = 8,
  height = 10,
  dpi = 300
)
```

```r
# 20.2 Uncertainty map
p_uncertainty <- ggplot() +
  geom_raster(data = uncertainty_df, aes(x = x, y = y, fill = value)) +
  geom_sf(data = study_sf_plot, fill = NA, colour = "black", linewidth = 0.45) +
  scale_fill_viridis_c(
    option = "magma",
    name = "Prediction\nuncertainty"
  ) +
  coord_sf(expand = FALSE) +
  theme_minimal(base_size = 14) +
  labs(
    title = "Prediction uncertainty",
    x = "Longitude",
    y = "Latitude"
  ) +
  theme(
    plot.title = element_text(face = "bold", size = 18, hjust = 0.5),
    legend.title = element_text(face = "bold"),
    legend.position = "right",
    panel.grid.major = element_line(colour = "grey85", linewidth = 0.2)
  )

ggsave(
  "outputs/figures/prediction_uncertainty.png",
  p_uncertainty,
  width = 8,
  height = 10,
  dpi = 300
```

```
)

# 20.3 Suitability class map
p_class <- ggplot() +
 geom_raster(data = class_df, aes(x = x, y = y, fill = value)) +
 geom_sf(data = study_sf_plot, fill = NA, colour = "black", linewidth = 0.45) +
 geom_point(
  data = points_df,
  aes(x = longitude_dd, y = latitude_dd),
  colour = "red",
  size = 1.0,
  alpha = 0.85
 ) +
 scale_fill_viridis_d(
  option = "D",
  name = "Suitability\nclass"
 ) +
 coord_sf(expand = FALSE) +
 theme_minimal(base_size = 14) +
 labs(
  title = "Habitat suitability classes",
  x = "Longitude",
  y = "Latitude"
 ) +
 theme(
  plot.title = element_text(face = "bold", size = 18, hjust = 0.5),
  legend.title = element_text(face = "bold"),
  legend.position = "right",
```

```
    panel.grid.major = element_line(colour = "grey85", linewidth = 0.2)
  )

ggsave(
  "outputs/figures/habitat_suitability_classes.png",
  p_class,
  width = 8,
  height = 10,
  dpi = 300
)

# 20.4 TSS binary map
p_binary <- ggplot() +
  geom_raster(data = binary_df, aes(x = x, y = y, fill = value)) +
  geom_sf(data = study_sf_plot, fill = NA, colour = "black", linewidth = 0.45) +
  geom_point(
    data = points_df,
    aes(x = longitude_dd, y = latitude_dd),
    colour = "red",
    size = 1.0,
    alpha = 0.85
  ) +
  scale_fill_manual(
    values = c(
      "Unsuitable" = "grey85",
      "Suitable" = "#2C7FB8"
    ),
    name = "Habitat\nclass"
```

```
  ) +
  coord_sf(expand = FALSE) +
  theme_minimal(base_size = 14) +
  labs(
    title = "Suitable habitat",
    x = "Longitude",
    y = "Latitude"
  ) +
  theme(
    plot.title = element_text(face = "bold", size = 18, hjust = 0.5),
    legend.title = element_text(face = "bold"),
    legend.position = "right",
    panel.grid.major = element_line(colour = "grey85", linewidth = 0.2)
  )

ggsave(
  "outputs/figures/suitable_habitat_TSS_threshold.png",
  p_binary,
  width = 8,
  height = 10,
  dpi = 300
)

################################################################
# 21. SAVE WORKSPACE AND FINAL CHECKS
################################################################

save(
```

```r
  bb_data,
  env_stack,
  env_mask,
  study_area,
  model_data_clean,
  predictor_names,
  cor_mat,
  collinearity_decision,
  fold_table,
  eval_by_fold,
  eval_table,
  ensemble_decision_fixed,
  final_models,
  map_list_raw,
  map_list_rescaled,
  selected_models,
  available_selected,
  selected_weights,
  ensemble_map,
  uncertainty_map,
  ensemble_class_quantile,
  tss_binary_map,
  area_class,
  area_binary,
  file = "outputs/black_bear_balanced_spatial_cv_workspace.RData"
)

msg("================ FINAL BALANCED SPATIAL CV CHECKS ================")
```

```
msg("Final predictors used:")
print(predictor_names)

msg("Fold counts:")
print(fold_table)

msg("Model evaluation summary:")
print(eval_table)

msg("Selected ensemble models:")
print(available_selected)

msg("Area by suitability class:")
print(area_class)

msg("Area by TSS binary class:")
print(area_binary)

msg("Analysis completed successfully.")
msg("Outputs saved in outputs folder.")
msg("Log saved at: ", log_file)
```

Our R code uses the following R packages:

1. terra (version 1.8-93)

Hijmans, R. J. (2020). terra: Spatial data analysis. *CRAN: Contributed packages*.

2. sf (version 1.0-24)

Pebesma, E. (2018). Simple features for R: standardized support for spatial vector data.

3. readr (version 2.1.5)

Wickham, H., Hester, J., & Bryan, J. (2018). Readr: Read rectangular text data.

4. dplyr (version 1.2.1)

Wickham, H., François, R., Henry, L., Müller, K., & Vaughan, D. (2023). dplyr: A grammar of data manipulation.

5. tidyr (version 1.3.1)

Wickham, H., Vaughan, D., & Girlich, M. (2020). Tidyr: Tidy messy data. *R package version*, *1*(2), 397.

6. pROC (version 1.19.0.1)

Robin, X., Turck, N., Hainard, A., Tiberti, N., Lisacek, F., Sanchez, J. C., & Müller, M. (2011). pROC: an open-source package for R and S+ to analyze and compare ROC curves. *BMC bioinformatics*, *12*(1), 77.

7. PRROC (version 1.4)

Grau, J., Grosse, I., & Keilwagen, J. (2015). PRROC: computing and visualizing precision-recall and receiver operating characteristic curves in R. *Bioinformatics*, *31*(15), 2595-2597.

8. randomForest (version 4.7-1.2)

Breiman, L. (2001). Random forests. *Machine learning*, *45*(1), 5-32.

9. gbm (version 2.2.3)

Ridgeway, G. (2006). gbm: Generalized boosted regression models. *R package version*, *1*(3), 55.

10. mgcv (version 1.9-3)

Wood, S. N. (2025). Generalized additive models. *Annual Review of Statistics and Its Application*, *12*(1), 497-526.

11. e1071 (version 1.7-16)

Meyer, D., Dimitriadou, E., Hornik, K., Weingessel, A., & Leisch, F. (2025). Misc Functions of the Department of Statistics, Probability Theory Group (Formerly: E1071), TU Wien [R package e1071 version 1.7-17]. *CRAN: Contributed Packages*, *10*.

12. maxnet (version 0.1.4)

Phillips, S. (2021). maxnet: Fitting 'Maxent' species distribution models with 'glmnet'. R package version 0.1.4. https://CRAN.R-project.org/package=maxnet

13. ggplot2 (version 4.0.0)

Wickham, H. (2016). Getting Started with ggplot2. In *ggplot2: Elegant graphics for data analysis* (pp. 11-31). Cham: Springer International Publishing.

14. viridis (version 0.6.5)

Garnier, S., Ross, N., Rudis, B., Filipovic-Pierucci, A., Galili, T., O'Callaghan, A., ... & Chen, J. J. (2023). sjmgarnier/viridis: CRAN release v0. 6.3. *Zenodo*.
